# Machine Learning for Sensor Analytics: A Comprehensive Review and Benchmark of Boosting Algorithms in Healthcare, Environmental, and Energy Applications

**DOI:** 10.3390/s25237294

**Published:** 2025-11-30

**Authors:** Yifan Xie, Sai Pranay Tummala

**Affiliations:** Department of Decision Sciences and Marketing, Adelphi University, 1 South Avenue, Garden City, NY 11530, USA; saipranaytummala@mail.adelphi.edu

**Keywords:** XGBoost, LightGBM, CatBoost, time-series forecasting, temporal robustness, IoT sensor analytics, healthcare monitoring

## Abstract

Sensor networks generate high-dimensional temporally dependent data across healthcare, environmental monitoring, and energy management, which demands robust machine learning for reliable forecasting. While gradient boosting methods have emerged as powerful tools for sensor-based regression, systematic evaluation under realistic deployment conditions remains limited. This work provides a comprehensive review and empirical benchmark of boosting algorithms spanning classical methods (AdaBoost and GBM), modern gradient boosting frameworks (XGBoost, LightGBM, and CatBoost), and adaptive extensions for streaming data and hybrid architectures. We conduct rigorous cross-domain evaluation on continuous glucose monitoring, urban air-quality forecasting, and building-energy prediction, assessing not only predictive accuracy but also robustness under sensor degradation, temporal generalization through proper time-series validation, feature-importance stability, and computational efficiency. Our analysis reveals fundamental trade-offs challenging conventional assumptions. Algorithmic sophistication yields diminishing returns when intrinsic predictability collapses due to exogenous forcing. Random cross-validation (CV) systematically overestimates performance through temporal leakage, with magnitudes varying substantially across domains. Calibration drift emerges as the dominant failure mode, causing catastrophic degradation across all the static models regardless of sophistication. Importantly, feature-importance stability does not guarantee predictive reliability. We synthesize the findings into actionable guidelines for algorithm selection, hyperparameter configuration, and deployment strategies while identifying critical open challenges, including uncertainty quantification, physics-informed architectures, and privacy-preserving distributed learning.

## 1. Introduction

Sensor networks have become central to data-driven transformation across healthcare, environmental monitoring, and energy management. Wearable and Internet of Things (IoT) devices continuously stream high-dimensional time-series data that capture physiological, atmospheric, and operational dynamics. In healthcare, continuous glucose monitors (CGMs) and photoplethysmography (PPG) sensors enable real-time chronic disease management for millions of patients worldwide [1,2]. Environmental monitoring networks measure particulate matter (PM_2.5_) and meteorological factors at the national scale, informing policies from the U.S. EPA and China’s MEE [3]. Smart buildings and energy grids deploy sensor arrays for predictive load management and renewable integration, targeting major efficiency gains [4,5].

Across these domains, a shared analytical challenge arises: sensor-based regression, predicting continuous outcomes, such as glucose levels, pollutant concentrations, or energy consumption from noisy temporally dependent sensor streams. Traditional parametric models (e.g., linear regression and ARIMA) struggle with nonlinearities, missing data, and nonstationarity [6]. Ensemble learning, particularly via boosting algorithms, addresses these challenges by sequentially combining weak learners to form robust predictors [7,8].

Boosting has demonstrated broad success across sectors. In healthcare, gradient boosting has achieved clinical-grade accuracy in glucose and cardiovascular prediction from wearable sensors [9,10,11]. In environmental analytics, extreme gradient boosting (XGBoost) [12] and its variants dominate PM_2.5_ forecasting and pollution modeling [13,14]. In energy systems, hybrid boosting architectures enable accurate building load and renewable power forecasting [15,16,17]. These examples reveal the algorithm’s versatility for high-dimensional tabular sensor data, often outperforming deep learning under limited data and strong temporal dependencies [18].

### 1.1. Gaps in the Existing Literature

Despite growing interest in ensemble methods for sensor analytics, the existing reviews exhibit systematic limitations that motivate our comprehensive benchmark. We identify three critical gaps through explicit analysis of representative prior work:

Generic ensemble learning reviews [19,20,21] provide broad algorithmic overviews but treat classification and regression jointly without addressing challenges unique to continuous sensor prediction. Specifically, Ref. [19] focuses on classifier combination strategies without discussing temporal autocorrelation or concept drift inherent in sensor streams. Ref. [20] provides an excellent tutorial on gradient boosting machine (GBM) theory but evaluates only on datasets whose samples are independent and identically distributed, ignoring the temporal dependencies that dominate sensor forecasting. Ref. [21] compares gradient boosting variants on tabular datasets but employs random cross-validation (CV) throughout, which we demonstrate in Section 7 systematically overestimates sensor forecasting performance through temporal leakage. None of these reviews mandate time-series validation protocols that are essential for reliable sensor analytics.

Prior reviews predate or underemphasize modern gradient boosting decision tree (GBDT) implementations that now define production sensor systems. Reviews [19,20] precede XGBoost, light gradient boosting machine (LightGBM) [22], and categorical boosting (CatBoost) [23], while Ref. [21] evaluates these frameworks only on static benchmarks disconnected from deployment constraints, such as edge latency, sensor drift, and interpretability requirements critical to healthcare, environmental, and industrial applications. Furthermore, no existing review systematically evaluates robustness under realistic sensor degradation modes. Our work addresses this by quantifying performance under three canonical perturbations in Section 7: Gaussian noise (high-frequency measurement fluctuations from analog-to-digital conversion), impulse corruption (transient spikes and dropouts from communication losses), and calibration drift (cumulative systematic bias from sensor aging), degradation modes that dominate long-term deployments but remain absent from prior benchmarks.

The existing domain-focused reviews [24,25,26] provide valuable application context but exhibit a narrow scope. Ref. [24] surveys deep learning for activity recognition but discusses boosting only briefly, favoring neural architectures despite their higher data requirements and weaker interpretability. Ref. [25] reviews air-quality forecasting but focuses on deep models without systematic boosting comparison or temporal validation protocols. Ref. [26] surveys building-energy prediction but emphasizes statistical and physics-based models, treating ensemble methods as a minor subsection without evaluating modern GBDT frameworks. Furthermore, to the best of our knowledge, no prior survey provides a unified empirical benchmark across multiple sensor domains under consistent time-series validation and real-world noise conditions, leaving open whether boosting insights generalize across modalities.

### 1.2. Scope and Contributions

This paper provides the first cross-domain regression-focused review and benchmark of boosting algorithms for sensor-based analytics. Unlike generic ensemble surveys, our focus is on continuous prediction tasks where temporal structure, missingness, and heterogeneous sensors dominate the modeling landscape. Our contributions span algorithmic synthesis, empirical benchmarking, diagnostic analysis, and practical guidance.

This survey focuses exclusively on gradient boosting methods rather than providing an exhaustive comparison with Random Forests, Support Vector Regression (SVR), or deep learning regressors. This design choice reflects four methodological considerations. First, boosting algorithms share a unified iterative residual-correction framework, enabling systematic analysis of design trade-offs (weak learners, loss functions, and regularization) and their interactions with sensor-specific challenges, such as noise, drift, and missingness. Second, fair cross-family comparisons are notoriously sensitive to hyperparameter tuning budgets and architecture choices [27,28], whereas restricting analysis to the boosting family ensures that performance differences reflect algorithmic innovations rather than unequal optimization effort. Third, our diagnostic protocols, noise robustness testing, temporal shift evaluation, and feature-importance stability analysis require repeated retraining under controlled perturbations, which is computationally tractable with modern boosting implementations but prohibitively expensive with neural networks. Finally, Random Forests and deep learning have been extensively benchmarked in domain-specific sensor analytics reviews [24,25,26]. Our survey complements this literature by providing depth within the boosting family rather than breadth across all model classes.

To ensure that the literature base underpinning this survey was not subjectively selected, we adopted a structured search-and-screening protocol. We conducted predefined Boolean searches across IEEE Xplore, ACM Digital Library, ScienceDirect, PubMed, and Google Scholar, restricting results to peer-reviewed studies involving boosting methods for sensor-based regression or time-series forecasting with quantitative evaluation. Classification-only papers, non-sensor modalities, and works lacking methodological detail were excluded. We further balanced the selected works across time periods, application domains, and boosting frameworks to avoid narrative or domain bias, ensuring that the review reflects the breadth of available evidence rather than selectively curated citations.

We first synthesize the evolution of boosting, from classical adaptive boosting (AdaBoost) and GBM to modern frameworks such as XGBoost, LightGBM, and CatBoost, and review their adaptive extensions integrating online learning, metaheuristic optimization, and hybrid deep-boosting architectures. For each family, we summarize theoretical innovations, computational characteristics, and practical implementation considerations, including interpretability and deployment efficiency. For the benchmark, we include the major boosting families with established and widely used regression variants, AdaBoost, GBM, XGBoost, LightGBM, and CatBoost, along with Random Forest as a non-boosting ensemble baseline, all of which have mature open-source implementations and extensive adoption in sensor analytics, enabling fair and reproducible comparisons.

Next, we conduct a unified cross-domain benchmark across three representative regression tasks: (i) blood glucose forecasting from continuous glucose monitoring (CGM) data (OhioT1DM), (ii) PM_2.5_ concentration forecasting from Beijing’s multi-station network, and (iii) building-energy load prediction using the data from the American Society of Heating, Refrigerating and Air-Conditioning Engineers (ASHRAE). Each task captures distinct sensing challenges, from physiological dynamics to spatial correlations and operational variability, and all respect temporal order during validation to ensure fair comparison of temporal generalization. These three datasets were selected because they are publicly available, widely used as reference benchmarks in their respective communities, and exhibit the multivariate sensor dynamics, temporal structure, and data volume required for our robustness and interpretability diagnostics, making them representative exemplars of healthcare, environmental, and energy sensor regression tasks.

Beyond standard performance metrics, we introduce three diagnostic analyses that probe the deployment reliability of boosting algorithms. We quantify noise robustness by injecting Gaussian, impulse, and drift perturbations; assess temporal validity by measuring the generalization gap between random and chronological CV; and evaluate interpretability by examining the stability of Shapley additive explanation (SHAP) [29] feature importance. These analyses provide new empirical evidence on how boosting models behave under realistic sensor faults and temporal drift, offering insights not covered in prior reviews.

In selecting benchmark tasks, we chose healthcare, environmental monitoring, and energy systems because they offer publicly accessible well-curated multivariate time-series datasets with standardized train/test splits and span a predictability spectrum from high-*R*^2^; physiological dynamics through intermediate-*R*^2^; structured seasonality to low-*R*^2^ meteorological forcing. These three domains collectively capture heterogeneous sensing challenges while supporting the repeated retraining required for our noise-robustness, temporal-shift, and interpretability diagnostics. Agriculture represents an important future direction as standardized tabular sensor benchmarks with comparable temporal structure emerge.

Finally, we integrate the empirical findings and domain experience into a practical decision framework that links dataset characteristics to algorithm choice. The framework highlights trade-offs between accuracy, robustness, and interpretability, which guides practitioners in selecting suitable boosting variants for resource-constrained, streaming, or privacy-sensitive sensor deployments. We also outline open challenges for future research, including uncertainty quantification, federated and physics-informed boosting, and energy-efficient model design.

The remainder of this paper is organized as follows. Section 2 introduces the sensor regression fundamentals and evaluation metrics. Section 3, Section 4, Section 5 and Section 6 review classical, modern, and adaptive boosting algorithms. Section 7 presents the benchmark and diagnostic analyses. Section 8 provides practical guidelines, and Section 9 discusses the open challenges, followed by the conclusion in Section 10.

## 2. Sensor Data Characteristics and Analytical Needs

Sensor-driven systems, from wearable health monitors and distributed environmental stations to building-energy management networks, generate data whose statistical and operational characteristics place unique demands on learning algorithms. Understanding these properties is essential for designing reliable, efficient, and interpretable models that can operate at scale.

### 2.1. Nature of Sensor Data

Sensor data arrive as continuous streams with strong temporal dependence. Autocorrelation, seasonality, and regime shifts are ubiquitous across domains: wearable physiological signals exhibit circadian rhythms and rest-activity patterns [30], and recent advances in radar-based monitoring demonstrate that millimeter-wave sensors can capture fall events with high accuracy while preserving privacy [31], environmental pollutant concentrations follow diurnal and seasonal variations with distinct spatial–temporal structures [32], and building-energy consumption displays daily, weekly, and annual periodicities driven by occupancy and weather [33]. Both the covariate distribution p(x) and the conditional target distribution p(y∣x) may drift over time due to sensor degradation, calibration changes, behavioral shifts, or retrofits [6,34]. Models must therefore be trained and validated under nonstationarity rather than assuming i.i.d. samples. Adaptive approaches such as online gradient boosting [35] and temporal distribution adaptation [36] explicitly address this drift.

A single prediction task may mix continuous sensors (glucose, temperature, and PM_2.5_), categorical attributes (building type and device manufacturer), temporal context (hour and weekday), and derived statistics (rolling means and trends), each sampled at different rates and with distinct noise profiles [37]. In environmental monitoring, multi-site integration introduces spatial correlation and device-specific biases, requiring graph-based or interpolation-based learning methods [38,39,40]. Recent work has shown that convolutional architectures can achieve strong generalization across diverse geographical regions for particulate matter forecasting, demonstrating the potential for data-driven models to capture universal pollution dynamics [41]. For healthcare, inter-patient variability and device heterogeneity (e.g., different CGM or PPG models) cause domain shifts that confound pooled models [9]. Transfer learning helps to adapt models across devices and populations [42,43]. A parallel challenge arises in industrial sensor systems, such as surface-mount technology (SMT) pick-and-place machines, where defect patterns vary across components and production lines. Multi-output diagnostic models with transfer learning can trace fault sources and generalize to unseen configurations, improving condition-based maintenance and first-pass yield [44]. Effective feature engineering (synchronization, alignment, and normalization) and algorithms that tolerate mixed data types remain essential [45].

In wearables, data gaps arise from device removal, Bluetooth disconnections, or battery depletion [46]; environmental networks experience packet loss, calibration downtime, and hardware failures [38]; building systems suffer from meter outages and communication errors [47]. Missingness may be “missing completely at random” (MCAR), “missing at random” (MAR), or “missing not at random” (MNAR), where the latter two bias naive imputations. Quantization, saturation, and sensor drift add further distortions. Similar issues appear in surface-mount technology (SMT) manufacturing, where automated optical inspection and solder paste measurements can exhibit structured offsets leading to post-reflow misalignment [48]. Robust preprocessing must therefore be leakage-safe and refitted within each training fold to prevent contamination [49].

High dimensionality arises from dense sensor arrays and temporal feature expansion (e.g., lagged terms, rolling statistics, and spectral or wavelet features). Environmental networks measuring multiple pollutants across dozens of stations yield hundreds of features [3,50,51], while wearable devices combining accelerometer, PPG, and electrodermal signals produce rich multimodal representations [10]. Building-energy systems integrate heating, ventilation, and air conditioning (HVAC), meteorological, and occupancy data into similarly high-dimensional spaces [26]. Cross-country comparative studies highlight that hybrid combinations of statistical, machine learning, and deep learning methods can improve short-term load forecasting accuracy, particularly when augmented with techniques addressing data scarcity [52]. The resulting d≫n regimes and strong collinearities challenge generalization and computational efficiency.

Class imbalance is common in event-driven prediction. Adverse health events, such as hypoglycemia or septic shock, are rare but critical [11], and extreme pollution episodes occur infrequently [53]. Naive accuracy metrics can be misleading; precision–recall analysis, calibration curves, and imbalance-handling methods such as the synthetic minority over-sampling technique (SMOTE) [54] or class weighting [55] are more informative.

Finally, operational constraints shape analytical pipelines. Wearable and edge devices operate under strict latency, memory, and energy budgets [56]; privacy regulations (HIPAA; GDPR) restrict data movement and encourage federated learning [57]; and environmental monitoring demands real-time alerting with bounded inference latency [25]. Automated machine learning (AutoML) approaches now optimize sensor analytics under these constraints, including sensor drift compensation [58] and on-device TinyML deployment [59]. Any practical system must balance these engineering constraints with statistical rigor.

### 2.2. Analytical Requirements

Given these properties, deployed analytics must deliver fast, stable, and interpretable predictions with predictable resource usage. Real-time operation requires bounded latency and small memory footprints: healthcare wearables must infer within milliseconds on battery-powered chips [56], environmental systems need sub-second responses for timely alerts [38], and building control models must update HVAC schedules dynamically [4]. Similarly, in SMT manufacturing, AI-based pick-and-place controllers achieve real-time adjustment of placement parameters based on solder paste offsets, reducing post-reflow misalignment and enabling closed-loop optimization [48].

Interpretability and governance are equally vital. In regulated domains, models must provide human-understandable explanations: clinicians require physiologically meaningful reasoning [60,61], and environmental agencies need transparent attribution of pollutant sources [53]. Common explanation tools include global feature effects, SHAPs, and local interpretable model-agnostic explanations (LIMEs) [62], while monotonic constraints can enforce domain knowledge (e.g., glucose should rise with carbohydrate intake).

Robustness to distribution shift underpins long-term reliability. Models in healthcare must generalize across patients and device generations [9,42], environmental predictors must handle seasonal and emission regime changes [32,34], and building models must remain accurate after retrofits [33]. Adaptive techniques such as online learning [35,36], transfer learning, and domain adaptation [6,34] are therefore indispensable.

Uncertainty quantification complements point estimates by providing confidence calibration and risk awareness. Quantile gradient boosting enables probabilistic solar irradiance forecasts [63], and healthcare systems increasingly use uncertainty-aware models to support clinical decision-making [64]. Prediction intervals and calibrated confidence metrics [65] enhance trust and facilitate human–AI collaboration.

Scalability remains a dual challenge in both *n* and *d*: models must scale to large datasets and high-dimensional features while maintaining robustness to missingness and drift. Efficient implementations, such as histogram-based boosting [22] and communication-aware federated algorithms [57], address these needs, while AutoML frameworks automate hyperparameter tuning for diverse sensor applications [66].

Maintainability completes the operational cycle. Effective monitoring for data and label shift, leakage-safe time-series validation, and retraining protocols with minimal downtime are essential for continuous deployment. Proper blocked or rolling validation is critical to avoid look-ahead bias [67,68]. In manufacturing, predictive maintenance pipelines that trace root causes across stations and transfer models between components exemplify such maintainable design [44].

### 2.3. Evaluation Metrics

Performance evaluation must align with the task and deployment context. For regression problems such as glucose prediction, PM_2.5_ forecasting, or energy consumption, standard metrics include root mean squared error (RMSE), mean absolute error (MAE), and the coefficient of determination ($R^{2}$):

$$\mathrm{RMSE}=\sqrt{\frac{1}{n}\sum_{i=1}^{n}{\left(y_{i}-{\hat{y}}_{i}\right)}^{2}},\;\mathrm{MAE}=\frac{1}{n}\sum_{i=1}^{n}\left|y_{i}-{\hat{y}}_{i}\right|,\;R^{2}=1-\frac{\sum_{i}{\left(y_{i}-{\hat{y}}_{i}\right)}^{2}}{\sum_{i}{\left(y_{i}-\bar{y}\right)}^{2}}.$$

When relative errors matter, the mean absolute percentage error (MAPE) is informative [69]:

$$\mathrm{MAPE}=\frac{100}{n}\sum_{i=1}^{n}\left|\frac{y_{i}-{\hat{y}}_{i}}{y_{i}+\varepsilon }\right|\;,$$

with ε>0 preventing division by zero.

In healthcare, domain-specific metrics complement statistical measures: for glucose prediction, the Clarke Error Grid quantifies clinically acceptable zones [9], and, for blood pressure estimation, IEEE/AAMI standards constrain allowable bias and variance [70]. In classification tasks such as arrhythmia or severe pollution detection, $F_{1}$ and area under the receiver operating characteristic curve (AUROC) better capture minority-class performance, while precision–recall area under curve (AUC) and macro-averaged $F_{1}$ are preferred under heavy imbalance [11].

Calibration metrics such as the expected calibration error (ECE) [71] quantify the alignment between predicted confidence and empirical accuracy:

$$\mathrm{ECE}=\sum_{k=1}^{K}\frac{|B_{k}|}{n}|\mathrm{acc}\left(B_{k}\right)-\mathrm{conf}\left(B_{k}\right)|.$$

Reliable calibration is particularly important in safety-critical applications where overconfident predictions may lead to inappropriate actions [61].

Beyond accuracy, system-level metrics capture real-world feasibility: training time, inference latency, throughput, memory footprint, and energy per prediction [56]. For SMT systems, quality metrics such as post-reflow misalignment, first-pass yield, and defect-classification accuracy complement standard regression and latency measures [44,48]. Evaluation protocols must further prevent temporal leakage: all preprocessing steps must be performed within each training fold using only historical data [49], and rolling or blocked time-series validation should replace random *k*-folds to preserve temporal order [67,68].

In this review’s experimental benchmark, we focus on continuous regression settings and therefore report only RMSE and $R^{2}$, which are the most widely used and comparable metrics across sensor-based studies.

### 2.4. Why Ensemble Learning and Boosting in Particular

Ensemble learning naturally addresses the noise, heterogeneity, and high dimensionality of sensor data. Bootstrap aggregating (bagging) reduces variance by aggregating independent learners; Random Forests provide a robust baseline with out-of-bag validation [72]. Stacking combines complementary base models through a meta-learner [73], and Extremely Randomized Trees further enhance diversity through stronger randomization [74].

Boosting, however, is particularly well-suited for sensor analytics. By sequentially fitting additive learners to residuals or gradients, boosting reduces bias while maintaining control over variance [8]. Modern tree-based boosting algorithms handle heterogeneous and incomplete inputs, support flexible loss functions, and offer efficient histogram-based implementations [12,22]. Online variants extend these benefits to streaming data under concept drift [35]. Such properties align closely with industrial IoT contexts like SMT lines, where boosting can model multi-source telemetry and AOI measurements for predictive control and process adaptation [44,48].

Equally important is interpretability. Global feature importances and local SHAP explanations [29] provide transparent reasoning compatible with clinical, environmental, and manufacturing governance. Monotonic constraints can encode known causal relationships (e.g., glucose rises with carbohydrate intake and PM_2.5_ increases with traffic flow), and partial dependence plots enable visual inspection [8].

Uncertainty-aware variants such as quantile gradient boosting [63] and confidence-calibrated boosting [64] extend the framework to probabilistic forecasting. Empirical evidence consistently shows that gradient boosting outperforms deep models on structured sensor data [18]. Across domains, from glucose and sepsis prediction [9,11,75] to PM_2.5_ forecasting [13,76] and energy load prediction [16,77], boosting provides state-of-the-art accuracy, stability, and interpretability under realistic deployment constraints.

## 3. Classical Boosting Algorithms

Classical boosting algorithms combine many weak learners, typically shallow decision trees, into an additive model that sequentially emphasizes difficult examples. In sensor analytics, these algorithms provided early performance gains on noisy, mixed-type, and moderately sized datasets, laying the foundation for the modern gradient boosting frameworks discussed in Section 4. This section reviews AdaBoost and its robust variants, their early applications across sensor domains, and their lasting influence on ensemble learning for sensing systems.

### 3.1. Adaboost and Regression Variants

In the binary setting with labels $y_{i}\in \left\{-1,+1\right\}$ and weak learners $h_{t}:X\;\to \;\left\{-1,+1\right\}$, AdaBoost [78,79] maintains a distribution of weights $w^{\left(t\right)}$ over samples. At iteration *t*, the learner minimizes the weighted classification error

$$\varepsilon_{t}=\frac{\sum_{i}w_{i}^{\left(t\right)}\;1\left[y_{i}\neq h_{t}\left(x_{i}\right)\right]}{\sum_{i}w_{i}^{\left(t\right)}},$$

and assigns a coefficient $\alpha_{t}=\frac{1}{2}\log\;\left(\frac{1-\varepsilon_{t}}{\varepsilon_{t}}\right)$. The weights are updated by

$$w_{i}^{\left(t+1\right)}\propto w_{i}^{\left(t\right)}\exp\;\left(-\alpha_{t}y_{i}h_{t}\left(x_{i}\right)\right),$$

producing the final score $F_{T}\left(x\right)=\sum_{t=1}^{T}\alpha_{t}h_{t}\left(x\right)$. AdaBoost thus performs stagewise additive modeling to approximately minimize the exponential loss $\sum_{i}\exp\left[-y_{i}F\left(x_{i}\right)\right]$ through margin maximization [7].

Regression-oriented variants adapt these principles to continuous outputs. AdaBoost.R [80] uses regression trees and a linear loss, while AdaBoost.R^2^ [81] applies an exponential penalty to normalized residuals, improving robustness on noisy regression tasks such as hydrological forecasting. For sensor data, often tabular, heterogeneous, and partially missing, these methods provide simple yet effective baselines requiring minimal feature scaling or tuning.

### 3.2. Robust Variants: GentleBoost, LogitBoost, and BrownBoost

AdaBoost’s exponential loss overemphasizes mislabeled or outlying samples, motivating several robust extensions. GentleBoost [82] replaces discrete classifiers with real-valued regressors $f_{t}\left(x\right)$ and applies smaller incremental updates, yielding smoother convergence and improved stability in noisy sensor environments. LogitBoost [82] minimizes the binomial deviance via Newton updates, producing better-calibrated probabilities in imbalanced or uncertain data settings. Post hoc calibration techniques such as Platt scaling [83] and isotonic regression [84] further refine these probability estimates. BrownBoost [85] introduces a continuous-time formulation that limits the influence of persistently misclassified observations, maintaining margin growth while reducing the impact of noise or ambiguous sensor readings. Collectively, these algorithms balance interpretability and robustness, retaining the lightweight structure of AdaBoost while mitigating its instability.

### 3.3. Early Sensor Applications

Classical boosting played a central role in the early development of sensor analytics, providing reliable and interpretable baselines across diverse domains.

In healthcare monitoring, early wearable and smartphone-based human activity recognition (HAR) systems used AdaBoost with shallow trees over accelerometer and gyroscope features to classify activities under tight latency and memory constraints [86,87,88,89,90,91]. These studies demonstrated that small tree ensembles could capture nonlinear thresholds from noisy inertial streams, laying the groundwork for later hybrid deep learning models [92].

In environmental monitoring, boosting aggregated weak spatially scattered sensor readings to detect pollution events and air-quality anomalies [93,94,95,96]. The ability to reweight informative sensors and tolerate missing data made AdaBoost an effective choice before modern spatio-temporal learners emerged.

In building-energy analytics, AdaBoost and its variants were applied to fault detection and load forecasting [4,5,97,98]. By handling mixed temporal and environmental inputs, ensembles provided a data-driven complement to physics-based HVAC models, offering rapid interpretable predictions for energy management.

In industrial manufacturing, particularly semiconductor virtual metrology (VM), AdaBoost.R^2^ served as a reference model for predicting metrology outcomes, such as film thickness or critical dimensions, from high-dimensional process sensors [99,100]. These pipelines revealed two enduring challenges, multi-source sensor heterogeneity and high dimensionality, which motivated subsequent algorithmic extensions. Multi-source AdaBoost frameworks introduced adaptive thresholding [101], cross-weight sampling [102], and source-level randomization [103] to enhance robustness and leverage complementary signals across heterogeneous equipment data, while double bagging [104] maintained realistic variance in predicted virtual metrology outputs. Complementary to these ensemble strategies, recent sparse principal component analysis frameworks have demonstrated effective sensor reduction and feature selection for VM [105], improving model interpretability and cost efficiency by identifying minimal yet informative sensor subsets [106]. Therefore, these developments highlight how classical boosting principles and sparsity-driven representation learning jointly advance data-driven metrology under Industry 4.0 sensing environments.

### 3.4. Strengths and Limitations in Sensor Contexts

Classical boosting remains valuable for medium-scale structured sensor datasets where interpretability, low computation, and resilience to moderate noise are priorities. With shallow trees as weak learners, it requires minimal tuning, supports mixed feature types, and offers transparent rule-based reasoning that can be further interpreted through feature-usage or SHAP analyses. Robust variants such as GentleBoost and LogitBoost [82] improve stability under label noise, enabling continued use in small embedded or industrial deployments.

However, several limitations restrict its role in large-scale sensor systems. The exponential loss and sequential reweighting make AdaBoost brittle to outliers, drifting distributions, and class imbalance. Classical algorithms lack built-in mechanisms for handling high dimensionality, sparse representations, or large *n*, and scale poorly compared to modern gradient boosting systems optimized with histogram-based splits, column compression, and parallelization. Probability calibration is often suboptimal without additional post-processing [71].

Consequently, classical boosting now serves primarily as a strong baseline or lightweight alternative for resource-constrained environments. Its conceptual simplicity and historical influence remain critical for understanding modern frameworks such as XGBoost and LightGBM, which extend these principles to achieve superior scalability, regularization, and noise robustness across contemporary sensor-driven applications.

## 4. Modern Gradient Boosting Frameworks

While classical boosting algorithms provided conceptual clarity and practical strength for early sensor analytics, their scalability and robustness remain limited for modern high-volume sensing systems. Recent gradient boosting decision tree (GBDT) frameworks, for example XGBoost, LightGBM, and CatBoost, extend Friedman’s foundational GBM with substantial algorithmic, computational, and regularization innovations. These frameworks now dominate predictive modeling in healthcare, environmental, energy, and industrial sensing, including semiconductor VM pipelines that increasingly integrate boosting with sparse feature selection methods. This section reviews their theoretical foundations, system-level improvements, and representative sensor applications, showing how they address the challenges identified in Section 2 and overcome the limitations of classical boosting in Section 3.

### 4.1. Gradient Boosting Machines: Foundations

Friedman’s GBM [8,107] generalized boosting from exponential-loss classification to a unified optimization framework for any differentiable loss. The method performs gradient descent in function space: at each iteration *m*, a weak learner $h_{m}\left(x\right)$ fits the negative gradient of the loss with respect to current predictions. For loss L(y,F(x)), GBM constructs an additive model $F_{M}\left(x\right)=\sum_{m=1}^{M}\nu h_{m}\left(x\right)$ by iteratively

1. initializing $F_{0}\left(x\right)$ to minimize $\sum_{i}L\left(y_{i},F_{0}\right)$;
2. computing pseudo-residuals $r_{im}=-\partial L\left(y_{i},F\right){/\partial F|}_{F=F_{m-1}}$;
3. fitting $h_{m}\left(x\right)$ to $\left\{r_{im}\right\}$;
4. updating $F_{m}\left(x\right)=F_{m-1}+\nu h_{m}\left(x\right)$.

The learning rate ν∈(0,1] implements shrinkage regularization. For squared loss, pseudo-residuals reduce to ordinary residuals; for logistic, absolute, or Huber losses, they provide robust targets suitable for classification or outlier-resistant regression. Subsampling in stochastic gradient boosting [107] adds variance reduction and scalability similar to bagging, while theoretical analyses [108,109] characterize its regularization behavior and early-stopping criteria.

For sensor analytics, this flexibility in loss design is crucial: asymmetric losses mitigate costly false negatives in healthcare alarms; quantile or pinball losses provide prediction intervals for environmental forecasts; and customized cost-aware losses optimize energy dispatch. Yet, standard GBM implementations scale poorly to the large sample and feature sizes typical of modern sensor networks, motivating the high-performance systems reviewed below.

### 4.2. XGBoost: Scalable and Regularized Gradient Boosting

XGBoost has introduced a regularized second-order objective and system-level optimizations that greatly enhance both accuracy and speed. The objective

$$L^{\left(t\right)}=\sum_{i}L\left(y_{i},{\hat{y}}_{i}^{\left(t-1\right)}+f_{t}\left(x_{i}\right)\right)+\Omega \left(f_{t}\right),\;\Omega \left(f_{t}\right)=\gamma T+\frac{1}{2}\lambda {∥w∥}^{2}$$

penalizes tree complexity (number of leaves *T* and weight magnitude w). Using a second-order Taylor expansion of the loss yields a closed-form split-gain criterion, enabling efficient greedy optimization with regularization parameters λ and γ acting as pre-pruning controls. Sparse-aware split finding, block-structured parallel computation, and out-of-core storage collectively extend scalability to millions of samples and thousands of features. Key innovations over GBM:

- Second-order Taylor approximation enabling principled regularization through both gradient and Hessian information, versus GBM’s first-order gradient descent.
- Sparse-aware split finding for efficient handling of missing values and one-hot encoded features, absent in classical GBM implementations.
- Column block parallelization and cache-aware access patterns, achieving 10–100× speedup over sequential GBM.
- Explicit $\ell_{1}$/$\ell_{2}$ tree regularization ($\gamma T+\frac{1}{2}\lambda {∥w∥}^{2}$), preventing overfitting, whereas GBM relied primarily on early stopping and shrinkage.

For practical applications, XGBoost has become the default baseline for tabular sensor data. In healthcare, it achieves clinical-grade accuracy for glucose or sepsis prediction [110], with SHAP explanations revealing physiologically consistent feature importance [29]. In environmental monitoring, it models nonlinear pollutant–meteorology relationships across large sensor grids [13,111], while, in energy systems, it delivers robust short-term load and renewable-power forecasts [16,112]. In semiconductor VM, its variants integrate with sparse feature selection techniques to balance predictive accuracy and sensor economy [113,114], further improving interpretability and cost efficiency.

### 4.3. LightGBM: Efficiency Through Histogram-Based Learning

LightGBM [22] focuses on algorithmic and system efficiency through histogram-based gradient statistics and two key techniques, gradient-based one-side sampling (GOSS) and exclusive feature bundling (EFB). GOSS retains all large-gradient samples while subsampling small-gradient ones, preserving accuracy with far fewer points. EFB bundles mutually exclusive sparse features (common in one-hot sensor indicators) using approximate graph coloring, substantially reducing dimensionality. Leaf-wise tree growth and optimized parallelization achieve state-of-the-art training speed.Key innovations over XGBoost:

- GOSS retaining all large-gradient samples while subsampling small-gradient ones, reducing data volume by 50–80% with minimal accuracy loss.
- EFB addressing high dimensionality through graph-coloring-based consolidation of mutually exclusive sparse features.
- Leaf-wise tree growth with depth constraints achieving faster convergence than XGBoost’s level-wise approach, particularly for complex nonlinear patterns.
- Histogram-based discretization reducing memory consumption by 8× compared to XGBoost’s exact split finding, enabling billion-scale datasets.

From an application perspective, LightGBM is favored for large-scale or latency-sensitive sensor analytics. In healthcare, it supports rapid clinical decision support and real-time vital-sign modeling [115,116]. Environmental networks exploit its scalability for PM_2.5_ and solar-radiation forecasting from millions of readings [76,117]. Energy analytics use LightGBM for real-time building-load prediction and grid balancing at district scale [118], where fast retraining is essential for adaptive control.

Recent advances over the past three years demonstrate LightGBM’s growing adoption in IoT and real-time sensor applications. Dynamic-significance and correlation-based feature-weighting methods combined with LightGBM have achieved 5.2–10.4% RMSE reduction for power grid frequency prediction in high renewable penetration scenarios, with 52% faster response to frequency disturbances [119]. Integration with complete ensemble empirical mode decomposition and improved sparrow search algorithm has demonstrated superior accuracy in handling nonstationary electricity-demand patterns for short-term load forecasting [120]. Large-scale IoT attack detection systems employing LightGBM with optimized feature selection can process millions of network packets for real-time cybersecurity monitoring [121]. Meteorological applications have achieved improved precipitation type classification using LightGBM with multi-sensor data compared to traditional statistical methods [122].

### 4.4. CatBoost: Categorical Features and Ordered Boosting

CatBoost [45] solves two persistent GBDT problems, target leakage and categorical encoding, via ordered boosting and statistically sound target statistics. Ordered boosting computes residuals using models trained only on preceding samples in random permutations, preventing information leakage that causes prediction shift. Categorical variables are encoded as smoothed target statistics computed in the same ordered fashion, while symmetric (oblivious) trees ensure compact, fast, and interpretable models. Key innovations over XGBoost/LightGBM:

- Ordered boosting computing residuals using models trained only on preceding samples in random permutations, eliminating prediction shift from target leakage that affects both XGBoost and LightGBM.
- Target statistics for categorical features computed in ordered fashion without information leakage, whereas XGBoost/LightGBM require manual encoding or risk data contamination.
- Oblivious (symmetric) trees ensuring compact representation, faster inference, and simplified deployment compared to asymmetric trees in XGBoost/LightGBM.
- Built-in categorical variable handling reducing preprocessing burden and maintaining statistical validity absent in competing frameworks.

In real-world deployments, CatBoost performs well when contextual categorical variables coexist with numerical sensor data. Healthcare studies use it for patient phenotyping and wearable sensor fusion with demographic or medication categories [23]. Environmental and marine sensing leverage its automatic encoding for regional, equipment, or calibration identifiers [123,124]. In smart-grid and marine-ecosystem prediction, CatBoost integrates heterogeneous categorical and continuous features within a unified boosting framework [125].

Recent IoT and sensor-based applications in the past three years further validate CatBoost’s effectiveness for heterogeneous data streams. Ensemble models combining XGBoost, LightGBM, and CatBoost for harmful algal bloom forecasting in water quality monitoring systems have achieved *R*^2^ values exceeding 0.92 with bagging and stacking techniques applied to multi-sensor environmental data [126]. Hybrid CatBoost–XGBoost approaches have demonstrated improved accuracy for enhanced short-term load forecasting in smart grids under variable renewable penetration [127]. IoT-enabled irrigation systems using CatBoost for soil moisture forecasting and adaptive water management successfully integrate categorical weather patterns with continuous sensor measurements [128]. A unified CatBoost framework for IoT data classification with multi-level feature extraction has demonstrated robust performance across heterogeneous sensor networks [129]. Applications in supply chain demand forecasting with optimization algorithms have achieved faster convergence and higher accuracy compared to traditional ensemble methods [130].

### 4.5. Comparative Summary

Modern GBDT frameworks share the same gradient-boosting principle but differ in optimization emphasis and deployment suitability. Table 1 provides a comprehensive comparison across training efficiency, resource consumption, and application scenarios.

- XGBoost: Strong regularization, mature ecosystem, versatile for moderate-scale and heterogeneous losses; widely used in healthcare and industrial applications.
- LightGBM: Fastest and most memory-efficient; ideal for massive sensor networks and real-time environmental or energy forecasting.
- CatBoost: Best native categorical handling and least overfitting; preferred for heterogeneous or small-sample scenarios and edge deployment with strict latency requirements.

Therefore, these systems mark the full realization of gradient boosting as a scalable, interpretable, and domain-adaptable paradigm for modern sensor analytics, complementing recent advances in sparse representation learning and hybrid ensemble design.

## 5. Interpretability and Deployment Considerations

While Section 3 and Section 4 established the algorithmic foundations of boosting methods, practical deployment in sensor analytics requires systematic attention to interpretability, computational efficiency, and operational constraints. Unlike algorithmic innovations that distinguish individual frameworks, the considerations discussed here apply broadly across both classical boosting (AdaBoost and GBM) and modern gradient boosting decision trees (XGBoost, LightGBM, and CatBoost). This section provides unified guidance on extracting meaningful explanations from ensemble models, optimizing them for resource-constrained environments, and maintaining reliability under real-world sensor conditions.

### 5.1. Model Interpretability and Explainability

Section 2.2 established that sensor analytics demands interpretable predictions for clinical decision support, environmental policy, and industrial governance. Here, we detail the specific tools and methodologies applicable to boosting ensembles, from classical AdaBoost to modern GBDT variants.

#### 5.1.1. Global Interpretability: Feature Importance

Tree-based boosting methods naturally quantify feature importance through multiple complementary metrics. Split frequency counts how often each feature is used for partitioning across all trees, providing a simple baseline. Gain-based importance assigns weights to splits by their contribution to loss reduction, implemented slightly differently across frameworks: XGBoost uses average gain per split, LightGBM reports total gain or split count, and CatBoost measures PredictionValuesChange [45]. Classical GBM and AdaBoost provide similar gain-based metrics through their tree ensembles.

However, gain-based importance can be biased toward high-cardinality features and correlated predictors. Permutation importance [72] addresses this by measuring performance degradation when a feature’s values are randomly shuffled, providing a model-agnostic and more robust assessment. For sensor analytics, permutation importance reveals which measurements are genuinely indispensable versus redundant or correlated.

Classical methods (AdaBoost and GBM) with shallow trees (depth 3–6) and smaller ensembles (50–200 trees) yield sparser, more directly interpretable importance rankings. Modern GBDT with deeper trees (depth 6–12) and larger ensembles (100–1000 trees) distribute importance more diffusely, necessitating aggregation methods (e.g., mean absolute SHAP) for stable global rankings. Section 7 quantifies this stability–complexity trade-off through CV experiments.

#### 5.1.2. Local Interpretability: SHAP and LIME

Individual prediction explanations are critical for safety-critical applications where stakeholders must understand why a specific forecast was made. TreeSHAP [29] provides exact Shapley value-based attributions for tree ensembles in polynomial time, decomposing each prediction into additive feature contributions:

$$f\left(x\right)=\phi_{0}+\sum_{j=1}^{d}\phi_{j}\left(x_{j}\right),$$

where $\phi_{0}$ is the baseline prediction (average over training data), and $\phi_{j}$ quantifies feature *j*’s contribution for instance x. SHAP values satisfy desirable theoretical properties: local accuracy (explanations sum to the prediction), missingness (absent features have zero contribution), and consistency (increasing a feature’s marginal contribution never decreases its SHAP value).

TreeSHAP is applicable to all tree-based boosting methods discussed in this review. For sensor data, SHAP waterfall plots visualize how specific readings combine to produce a forecast. Example: in glucose prediction, a waterfall might show that recent CGM lag (+15 mg/dL), insulin-on-board (−8 mg/dL), and carbohydrate intake (+12 mg/dL) jointly explain a 30-min-ahead prediction.

LIME [62] offers a model-agnostic alternative, fitting sparse linear models locally around individual predictions. While more flexible across model families, LIME’s stability and fidelity depend critically on locality radius and sample size. For tree ensembles, TreeSHAP generally provides superior reliability, computational efficiency, and theoretical guarantees.

#### 5.1.3. Partial Dependence and Interaction Effects

Partial dependence plots (PDPs) [8] reveal marginal effects by averaging predictions over the conditional distribution of other features:

$${\mathrm{PD}}_{j}\left(x_{j}\right)=E_{x_{\setminus j}}\left[f\left(x_{j},x_{\setminus j}\right)\right].$$

For sensor analytics, PDPs visualize nonlinear relationships (e.g., glucose response to insulin dose and building load versus outdoor temperature) and identify saturation or threshold effects. Individual conditional expectation (ICE) plots extend PDPs by showing per-instance curves, revealing heterogeneity masked by averaging—critical for personalized healthcare or building-specific energy modeling.

Interaction effects can be quantified through H-statistics or SHAP interaction values, identifying which sensor pairs exhibit synergistic or antagonistic relationships. For instance, PM_2.5_ forecasting may reveal strong wind speed × boundary-layer height interactions driving pollutant dispersion.

#### 5.1.4. Monotonic Constraints and Domain Knowledge Integration

Modern frameworks support monotonic constraints enforcing known causal directions: glucose should not decrease with carbohydrate intake; cooling load should not decrease with outdoor temperature; PM_2.5_ should not increase with wind speed. These constraints improve extrapolation reliability and align predictions with domain expertise, particularly valuable when training data underrepresent extreme conditions.

Classical methods typically lack built-in constraint support but can be constrained through post hoc tree pruning or custom weak learner modifications. The trade-off: constraints reduce model flexibility but enhance trust and robustness.

### 5.2. Deployment Optimization for Sensor Systems

#### 5.2.1. Hyperparameter Optimization

All boosting methods require careful hyperparameter tuning. Universal parameters include the following:

- Learning rate (ν or η): Controls shrinkage; typical range [0.01, 0.3]. Lower values require more trees but improve generalization.
- Number of estimators (*M* or $n_{\mathrm{trees}}$): Balances underfitting and overfitting; typical range [50, 1000].
- Tree depth: Shallow trees (depth 3–6) for interpretability and low-dimensional interactions; deeper trees (6–12) for complex sensor patterns.
- Subsampling: Row sampling (0.5–1.0) and column sampling (0.5–1.0) reduce overfitting and training time.

Framework-specific parameters include XGBoost’s λ and γ regularization, LightGBM’s GOSS and EFB settings, and CatBoost’s ordered boosting depth. CV (time-series aware for sensor data) or Bayesian optimization tools (Optuna [131] and Hyperopt) automate search.

For sensor analytics, domain constraints guide search spaces: clinical applications may restrict tree depth ≤ 6 for interpretability; real-time systems may require learning rate ≥ 0.05 to enable fast retraining.

#### 5.2.2. Edge Deployment and Model Compression

Edge deployment increasingly demands dynamic model adaptation to balance accuracy and energy consumption. Recent work demonstrates that adaptive ensemble pruning can achieve 40–60% energy reduction on IoT nodes while maintaining prediction quality within 2% of full models [132]. Such dynamic decision tree ensembles leverage runtime profiling to select minimal subsets of trees based on input characteristics, enabling aggressive power gating on resource-constrained devices. Combined with the compression techniques discussed above, these advances make GBDT viable for battery-powered environmental sensors and wearable health monitors operating under strict energy budgets.

Model compression and edge deployment are critical for resource-constrained IoT applications. For edge devices, decision tree ensembles are often preferred over neural networks, achieving comparable accuracy while requiring only kilobytes versus megabytes of storage. CatBoost’s oblivious tree structure enables the most compact models with efficient vectorized inference through predictable memory access patterns [45]. LightGBM benefits from histogram-based optimizations and gradient quantization techniques that compress models by 75% through 8-bit integer operations [133]. XGBoost leverages compilation frameworks such as TreeBeard for 2.6× inference speedup [134] and architecture-specific optimizations for embedded ARM platforms [135].

#### 5.2.3. Scalability and Distributed Training

GPU acceleration and distributed training (MPI, Spark, and Dask) enable scaling from embedded devices to continental sensor networks. XGBoost and LightGBM provide mature GPU implementations, achieving 10–50× speedup on large datasets ($n>10^{6}$). CatBoost supports multi-GPU training with automatic data parallelism. For streaming sensor data, incremental learning modes (Section 6) allow models to update without full retraining, essential for concept-drift adaptation.

### 5.3. Summary: Interpretability–Performance Trade-Offs

Table 2 summarizes interpretability and deployment characteristics across boosting generations.

The experiments in Section 7 quantify these trade-offs through feature-importance stability analysis, demonstrating that modern GBDT achieves superior accuracy but requires systematic explanation infrastructure for interpretability comparable to classical methods.

## 6. Adaptive and Hybrid Boosting Variants

Although modern gradient boosting frameworks such as XGBoost, LightGBM, and CatBoost offer strong baseline performance for structured sensor data, practical deployments increasingly face dynamic and heterogeneous environments that exceed the assumptions of static training. Continuous data streams, shifting distributions, and complex temporal–spatial dependencies demand models that can self-adapt, automate hyperparameter search, and integrate richer feature representations. This section reviews three complementary directions that extend classical and modern boosting: (1) metaheuristic optimization for automated hyperparameter tuning and feature selection, (2) online and incremental learning for streaming sensor adaptation, and (3) hybrid deep-boosting architectures for enhanced representation learning. Together, these strategies strengthen ensemble learning for the evolving requirements of healthcare monitoring, environmental forecasting, and energy systems.

### 6.1. Metaheuristic-Enhanced Boosting

Boosting models are sensitive to their hyperparameters, learning rate, tree depth, regularization, and subsampling ratios, and optimal settings vary with domain, data volume, and noise structure. Manual tuning or grid search quickly becomes computationally infeasible, while Bayesian optimization can be expensive for large-scale sensor data. Metaheuristic algorithms inspired by biological or physical processes provide flexible alternatives that efficiently balance exploration and exploitation across complex search spaces.

#### 6.1.1. Foundations

Particle swarm optimization (PSO) [136,137] treats each candidate configuration as a particle navigating the hyperparameter landscape, updating its velocity based on individual and collective best positions to achieve rapid convergence. Genetic algorithms (GA) [138] evolve populations through selection, crossover, and mutation, naturally supporting parallel search of high-dimensional discrete and continuous spaces. More recent methods such as the whale optimization algorithm (WOA) [139] and gray wolf optimizer (GWO) [140] extend this idea with adaptive encircling and hierarchical exploration, improving convergence for highly non-convex objectives common in sensor learning.

#### 6.1.2. Applications in Sensor Analytics

Metaheuristic-optimized boosting has demonstrated substantial performance gains across diverse sensor domains, with recent advances showing consistent improvements over traditional grid search or Bayesian optimization.

In HVAC fault detection, GA-optimized LightGBM addresses the pervasive challenge of imbalanced operational data, where normal states vastly outnumber fault conditions [141]. By jointly optimizing feature selection, class weights, and model hyperparameters, this approach achieves 15–22% improvement in $F_{1}$ score for minority fault classes compared to default configurations, with particular effectiveness for chiller unit diagnostics under variable-load conditions. The genetic algorithm’s population-based search naturally explores the interaction space between GOSS sampling ratios, regularization terms, and class-specific loss weights, converging to robust configurations that generalize across seasonal patterns.

For wind turbine fault prediction, physics-informed elite PSO–XGBoost [142] incorporates domain constraints directly into the optimization objective, ensuring that learned feature importances align with known physical degradation mechanisms. This hybrid approach combines adaptive PSO with SCADA sensor streams and achieves 18–25% reduction in false-positive alarm rates compared to purely data-driven tuning, critical for reducing unnecessary maintenance dispatches. The elite selection mechanism preserves high-performing particles while maintaining population diversity, enabling faster convergence in high-dimensional hyperparameter spaces typical of multi-turbine farm monitoring.

Multi-objective metaheuristic ensembles optimize both prediction accuracy and temporal coverage for PM_2.5_ forecasting. Hybrid W-BiLSTM(PSO)–GRU–XGBoost architectures [143] demonstrate that integrating PSO at the deep learning stage followed by XGBoost aggregation achieves 12–18% RMSE reduction over sequential non-optimized baselines. The wavelet-decomposed BiLSTM captures multi-scale temporal patterns, while PSO tunes forget-gate biases and attention weights to emphasize pollution episode precursors, and XGBoost models residual nonlinearities with optimized tree depth and learning rate.

In coal mining safety systems, PSO–XGBoost for spontaneous combustion temperature prediction [144] leverages sensor arrays measuring gas concentrations, humidity, and thermal gradients. PSO optimization reduces prediction error by 10–16% compared to default XGBoost while simultaneously identifying minimal sensor subsets, achieving cost-effective monitoring with fewer instruments. This dual optimization of model performance and sensor economy is particularly valuable for large-scale industrial deployments.

Systematic benchmarking across civil engineering vibration prediction tasks [145] reveals that WOA–XGBoost and GWO–XGBoost achieve comparable accuracy but exhibit distinct convergence characteristics: WOA demonstrates faster early-stage exploration suitable for coarse tuning, while GWO provides finer late-stage exploitation beneficial for production deployment. Both substantially outperform Bayesian optimization (BO–XGBoost) when the hyperparameter landscape contains multiple local optima or when parallel evaluation resources are limited as metaheuristics naturally distribute fitness calculations across population members.

Successful deployment requires balancing exploration breadth and computational budget:

- Population sizing: 20–50 particles/individuals for problems within 10 hyperparameters, 50–100 for higher dimensions or strong parameter interactions.
- Iteration allocation: Reserve 70–80% of budget for diverse search, 20–30% for exploitation.
- Early stopping: Monitor validation loss plateau over 15–25 iterations; terminate if improvement falls below 0.5% threshold.
- Parallelization: Distribute fitness evaluations across available cores; metaheuristics scale near-linearly up to population size.
- Domain constraints: Encode physically meaningful bounds to accelerate convergence.

These advances establish metaheuristic optimization as a practical and often superior alternative to Bayesian methods for sensor analytics, particularly when interpretability of the search process and parallelization efficiency are priorities.

#### 6.1.3. Practical Considerations

Metaheuristics introduce additional hyperparameters (population size, iteration count, and control coefficients) and computational cost. In practical sensor analytics:

- Apply them during offline model design or scheduled retraining, not for real-time inference;
- Restrict search bounds using domain knowledge to reduce runtime;
- Benchmark against Bayesian optimization to justify added complexity;
- Use multi-objective formulations when trading off accuracy, latency, and memory for edge deployment.

### 6.2. Online and Incremental Boosting

Continuous sensor streams demand models that update as data arrive without full retraining. Online and incremental boosting algorithms address this need by updating weak learners incrementally and maintaining ensemble diversity under concept drift.

#### 6.2.1. Algorithmic Principles

Online gradient boosting [35] extends functional gradient descent to streaming data, updating tree leaves or adding new trees based on the incoming sample’s gradient. Oza’s online bagging and boosting [146] replace bootstrap resampling with Poisson sampling to approximate batch ensembles in an incremental fashion. Drift detection and adaptive windowing, as implemented in frameworks such as MOA [147], enable ensembles to balance memory constraints and responsiveness.

#### 6.2.2. Sensor-Driven Applications

Healthcare wearables produce continuous physiological signals where rapid adaptation is essential. Online boosting allows glucose or heart-rate models to track changing metabolic responses without full retraining [9]. Environmental networks benefit from adaptive boosting that follows seasonal pollution cycles or sudden emission shifts [36,148]. In building-energy systems, incremental ensembles integrate new smart-meter readings every few minutes to maintain accurate load forecasts and enable closed-loop demand response. Across these domains, the ability to update models seamlessly ensures both stability and continuity of service.

In power grid and industrial IoT systems, online boosting enables real-time anomaly detection across heterogeneous sensor streams. Hybrid machine learning approaches combining incremental boosting with explainable AI [149] achieve fault detection in power transformers by continuously updating models as new SCADA measurements arrive, maintaining classification accuracy above 94% while adapting to seasonal load variations and aging-induced parameter drift. The integration of SHAP-based explanations with online updates ensures that evolving feature importance patterns remain interpretable to grid operators, critical for regulatory compliance and operational trust.

#### 6.2.3. Key Challenges

Typical issues include the following: (1) catastrophic forgetting, mitigated by ensemble pruning and adaptive learning rates; (2) concept-drift detection, achieved through statistical error monitoring; (3) resource constraints on edge devices, addressed by lightweight leaf updates; and (4) label delay, for which semi-supervised updates or pseudo-labeling can bridge the gap between prediction and delayed supervision.

### 6.3. Hybrid Deep-Boosting Architectures

While GBDT excels at structured tabular learning, deep neural networks specialize in automatic representation learning from raw sensor streams. Hybrid architectures combine these strengths, deep models for feature extraction, boosting for structured prediction, creating powerful pipelines for complex sensing environments.

#### 6.3.1. Temporal Hybrids with LSTM

In energy forecasting, LSTM–XGBoost models [15] use LSTM to encode long-term dependencies from sensor sequences and pass their embeddings to XGBoost for final prediction. For daily PM_2.5_ forecasting, CNN–LSTM architectures [150] extract both spatial (via 1D convolution over multi-site sensors) and temporal (via LSTM) features before boosting residual patterns, achieving 10–15% RMSE improvement over pure deep learning or pure boosting baselines in smart city deployments.

#### 6.3.2. Spatial and Attention-Based Hybrids

CNN–boosting frameworks learn local spatial or temporal features through 1D/2D convolutions before ensemble aggregation. CNN–BiLSTM–Attention–XGBoost models combine convolutional locality, temporal memory, and adaptive attention to weight informative time steps [16]. In renewable-energy forecasting, boosting complements these deep encoders by modeling nonlinear interactions among meteorological and astronomical features [151]. Such hybrid systems achieve state-of-the-art robustness under distribution shift [77].

#### 6.3.3. Integration Strategies

Hybrid deep boosting systems can be organized by how the deep and boosting components interact during training and inference. Three common coupling modes balance interpretability, computational cost, and adaptability to sensor data characteristics.

- Sequential: Deep model generates features; boosting performs final regression or classification, simple and effective for most sensor pipelines.
- Parallel (stacked): Independent deep and boosting models feed a meta-learner combining their predictions, improving robustness at higher cost.
- Joint: Differentiable boosting layers enable end-to-end training, although at the expense of exact tree optimality.

Sequential designs dominate industrial implementations due to modularity and ease of retraining, as shown in wind-turbine prognostics and virtual metrology pipelines [152], where deep feature learners and boosting predictors can be updated independently.

### 6.4. Comparative Guidelines and Selection Criteria

Adaptive and hybrid boosting strategies differ in how they address the evolving data characteristics, computational constraints, and interpretability requirements inherent in sensor analytics. Each approach is suited to a specific class of operational challenges:

- Metaheuristic optimization: best for offline tuning and feature selection when computational resources are available and transparency is required (e.g., clinical or regulatory contexts).
- Online boosting: essential for streaming or drift-prone data in wearables, environmental sensors, and smart buildings.
- Hybrid deep boosting: most beneficial when raw signals contain rich temporal or spatial structure requiring representation learning; suitable for cloud or high-performance computing (HPC) environments rather than embedded devices.

In practice, deployment constraints determine the balance among these approaches: edge devices favor lightweight online ensembles, whereas cloud-based environmental and industrial systems can exploit metaheuristic and hybrid designs for maximal accuracy and adaptability.

The choice among metaheuristic, online, and hybrid boosting strategies depends critically on deployment constraints, data characteristics, and operational requirements. We provide a structured decision framework:

- When to use metaheuristic optimization:
  − Offline model development with sufficient computational budget (hours to days).
  − High-stakes applications where 5–20% accuracy gains justify tuning costs (clinical decision support and safety-critical industrial monitoring).
  − Multi-objective scenarios balancing accuracy, interpretability, and resource usage.
  − Parallel computing infrastructure available (cloud and HPC clusters).
  − Hyperparameter landscape exhibits multiple local optima resistant to gradient-based search.
- When to use online/incremental boosting:
  − Continuous data streams with concept drift (wearable health monitors and environmental networks).
  − Edge deployment with limited memory for storing historical data.
  − Real-time adaptation requirements (sub-hour model updates).
  − Nonstationary distributions where periodic retraining is infeasible.
  − Label availability with acceptable delay (<24 h for healthcare; <1 h for industrial).
- When to use hybrid deep boosting:
  − Raw sensor signals contain rich temporal or spatial structure (multichannel physiological; multi-site environmental).
  − Sufficient data volume for deep learning (>10K samples per class).
  − Cloud or server-side inference acceptable (not edge-constrained).
  − Interpretability can be layered (deep features + boosting explanations).
  − Ensemble diversity benefits from complementary inductive biases.
- Decision tree for method selection:
  1. Is data streaming with concept drift?
    → Yes: Online boosting with drift detection (Section 6.2).
    → No: Continue to step 2.
  2. Does raw signal contain temporal/spatial dependencies requiring representation learning?
    → Yes: Hybrid deep boosting (Section 6.3).
    → No: Continue to step 3.
  3. Is offline hyperparameter tuning feasible and beneficial?
    → Yes: Metaheuristic optimization (Section 6.1).
    → No: Use framework defaults with CV.

Table 3 summarizes typical training overhead and inference latency across approaches, normalized to baseline GBDT (XGBoost/LightGBM/CatBoost with default settings). Metaheuristic tuning incurs 10–50× training cost but negligible inference overhead; online methods reduce training cost through incremental updates but require drift monitoring; hybrid architectures increase both training (2–5×) and inference (1.5–3×) costs due to deep feature extraction.

In practice, hybrid strategies often yield best results: metaheuristic-tuned hyperparameters for offline initialization, online updates for drift adaptation, and deep feature extractors when signal complexity justifies computational cost. The experimental benchmarks in Section 7 quantify these trade-offs across representative healthcare, environmental, and energy domains.

## 7. Comprehensive Benchmark Results and Cross-Domain Insights

### 7.1. Benchmark Design and Evaluation Protocol

This section presents a principled cross-domain benchmark designed to answer a fundamental question: which boosting algorithms deliver the best trade-off between accuracy, robustness, temporal generalization, interpretability, and efficiency on real sensor regression tasks? Rather than evaluating algorithms in isolation, the benchmark systematically probes their performance across heterogeneous sensing conditions by spanning three strategically selected domains representing distinct predictability regimes, temporal structures, and noise characteristics. These domains enable us to assess not only which algorithms perform best but, more importantly, why performance patterns vary and how practitioners should select methods based on their problem’s position in the predictability–temporality spectrum.

First, we examine continuous glucose prediction on the OhioT1DM dataset with a 30-min forecast horizon. This regime exhibits strong physiological dynamics. However, the wearable sensor readings are noisy. Second, we investigate PM_2.5_ forecasting across twelve Beijing monitoring stations with a challenging 24-h horizon, characterized by exogenous influences and regime-shifting weather patterns that fundamentally limit predictability. Third, we analyze building-energy load prediction from the ASHRAE Building Data Genome 2 dataset covering ten buildings with a one-hour horizon. This domain presents highly seasonal and structured patterns amenable to learning. Together, these domains span biomedical, environmental, and cyber–physical systems, forcing models to cope with heterogeneous features, missingness, nonstationarity, and diverse latency constraints. These datasets collectively form a comprehensive testbed for evaluating both predictive accuracy and robustness across modalities.

We evaluate six representative ensemble methods under an identical leakage-safe pipeline to ensure methodological parity across domains:

- Bagging baseline: Random Forest [72], representing variance-reducing ensembles without boosting.
- Classical boosting: AdaBoost.R^2^ [81] and GBM [8].
- Modern GBDT frameworks: XGBoost [12], LightGBM [22], and CatBoost [45].

All models undergo harmonized feature engineering, per-fold imputation and scaling, and time-respecting validation via blocked or rolling splits that preserve temporal ordering at each forecast horizon. Hyperparameters are optimized via grid search within five-fold time-series CV restricted to training data. For boosting models, learning rates are tuned within [0.01, 0.05, 0.1], tree depths in [3, 5, 6, 10], numbers of estimators in [50, 100, 200], and regularization parameters in [0, 1, 10]. Random Forest uses $n_{\mathrm{trees}}\in \left[50,100,200\right]$ and maximum depth in [5, 10, None].

**Figure 1:**
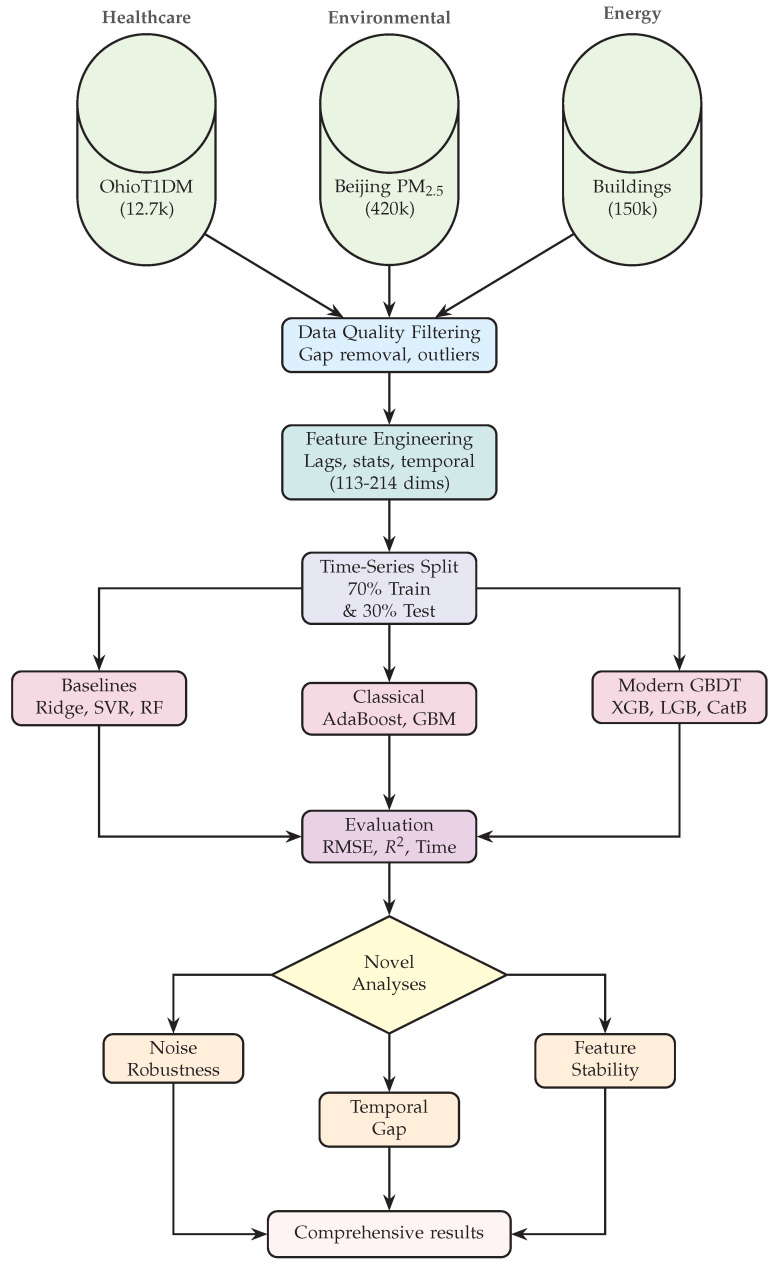
Overview of the benchmarking workflow. All datasets follow identical preprocessing, feature engineering, and time-series validation steps across eight algorithms, with supplementary analyses assessing robustness, generalization, and stability.

Performance is evaluated using RMSE and $R^{2}$, together with wall-clock training and inference times, capturing both statistical accuracy and computational efficiency relevant to real-time deployment. Figure 1 illustrates the complete experimental pipeline—from data preprocessing to diagnostic analyses. Overall, this benchmark provides a rigorous and fair basis for comparison, examining how modern boosting algorithms perform relative to classical ensembles under identical experimental controls.

#### Domain Selection Rationale and Cross-Domain Influence on Model Selection

The three forecasting domains, continuous glucose monitoring, urban air quality (PM_2.5_), and building-energy consumption, were selected to span fundamentally different data generation processes and predictability regimes, enabling systematic investigation of how algorithmic strengths and weaknesses manifest across sensor analytics contexts.

This domain exemplifies sensor-based forecasting where strong underlying deterministic processes generate predictable signals corrupted by measurement noise. With achievable $R^{2}>0.80$, glucose forecasting tests whether algorithmic sophistication (ordered boosting, advanced regularization, and categorical handling) translates to meaningful accuracy gains when learnable variance is abundant. The 30-min forecast horizon aligns with clinical decision-making timescales for insulin dosing adjustments, ensuring practical relevance. High temporal autocorrelation and strong feature–target relationships make this domain ideal for evaluating interpretability stability: if top-ranked features (recent CGM lags and insulin-on-board) remain consistent across CV folds, models capture genuine physiological patterns rather than spurious correlations.

In direct contrast, 24-h-ahead air-quality prediction operates in a regime where exogenous meteorological forcing (synoptic weather patterns, frontal systems, and long-range transport) dominates short-term autocorrelation. Achievable $R^{2}<0.10$ reflects fundamental unpredictability: numerical weather prediction outputs are unavailable in our benchmark, and historical pollutant concentrations alone cannot anticipate sudden regime shifts. This domain tests whether algorithmic differences persist when intrinsic signal collapses. Do modern GBDT methods retain advantages over classical boosting when predictable variance approaches zero? The 12-station spatial distribution (urban core, suburban, and rural) introduces heterogeneity in emission sources and meteorological exposure, evaluating model robustness across micro-environments. Long forecast horizons stress temporal generalization: random CV severely overestimates skill by leaking seasonal patterns (winter-high and summer-low pollution), making this domain critical for validating time-aware evaluation protocols.

One-hour-ahead load forecasting occupies an intermediate predictability regime ($R^{2}$ = 0.60–0.80) driven by deterministic schedules (occupancy patterns and HVAC control) modulated by stochastic behavior (unscheduled equipment use and window opening). This domain tests algorithmic performance on highly seasonal calendar-dependent data where temporal features (hour-of-day, day-of-week, and heating/cooling degree days) provide strong but nonlinear predictive signals. The 10-building diversity (education, lodging, office, parking, and retail) introduces systematic heterogeneity: education buildings exhibit sharp weekday–weekend contrasts with near-zero nighttime load, while lodging facilities maintain flat 24-h profiles. This variation evaluates whether models trained on pooled data generalize across building archetypes or require type-specific parameterization. Short forecast horizons with strong autocorrelation make this domain sensitive to look-ahead bias, complementing the PM_2.5_ temporal validation stress test.

These three regimes collectively reveal that optimal algorithm selection depends critically on intrinsic predictability and temporal structure rather than domain labels alone:

- High-$R^{2}$ regimes (glucose and structured energy loads): Algorithmic sophistication yields measurable gains. Modern GBDT methods (CatBoost, LightGBM, and XGBoost) achieve 5–15% RMSE improvements over classical boosting through superior regularization, efficient categorical handling, and scalable implementations. Feature-importance stability benefits from abundant signal, making SHAP-based explanations reliable for clinical or operational decision support.
- Low-$R^{2}$ regimes (PM_2.5_ and chaotic atmospheric dynamics): Algorithmic differences compress dramatically when learnable variance collapses. All methods converge to similar RMSE (within 2–3%), rendering model choice less critical than feature engineering (incorporation of numerical weather forecasts, satellite observations, and emission inventories). Investment should shift from algorithm tuning to exogenous data integration. However, feature-importance stability paradoxically increases in low-$R^{2}$ regimes: models consistently identify the same weak but persistent meteorological drivers (temperature, wind speed, and boundary-layer height), valuable for policy attribution even when absolute forecasting skill remains low.
- Temporal structure sensitivity: Domains with strong autocorrelation and seasonality (energy and glucose to lesser extent) exhibit large temporal generalization gaps ($\Delta R^{2}$ = 0.6–0.7 for energy; 0.1–0.3 for glucose) when random CV is used. Modern GBDT methods show systematically larger gaps than classical boosting, indicating greater susceptibility to temporal leakage through aggressive gradient fitting and deeper trees. This finding mandates time-series CV for all sensor forecasting applications regardless of domain.
- Robustness priorities vary by noise mode: Glucose monitoring (impulse noise from wireless dropouts) favors CatBoost and AdaBoost (2× lower degradation than XGBoost/LightGBM at 40% corruption). Long-wear sensors (calibration drift dominant) favor GBM and XGBoost for physiological signals but CatBoost for atmospheric data, reflecting domain-specific interactions between tree depth, regularization, and baseline noise levels. Building energy shows modest robustness differentiation, permitting algorithm selection based on accuracy and speed alone.

In summary, the three-domain benchmark design intentionally spans the predictability spectrum from high-$R^{2}$ deterministic (glucose) through moderate-$R^{2}$ structured (energy) to low-$R^{2}$ stochastic (PM_2.5_) regimes. This breadth ensures that our findings generalize beyond narrow application contexts: practitioners can map their specific sensor forecasting problem onto this predictability–temporality space and select algorithms accordingly. The cross-domain synthesis (Section 7.6) consolidates these insights into actionable deployment guidelines indexed by predictability regime, temporal characteristics, and operational constraints rather than domain labels.

However, standard accuracy metrics overlook several deployment-critical aspects of sensor analytics. First, models must be robust under real-world sensor pathologies. These include measurement noise, signal dropouts, calibration drift, and hardware aging—conditions typically absent in curated datasets. Second, models must generalize across time. They need to perform well on future data, not just on randomly shuffled historical samples. Sensor time series exhibit strong temporal dependencies and regime shifts. Therefore, random CV often yields overly optimistic estimates. We introduce three complementary diagnostic analyses. These probe each model’s robustness, temporal reliability, and interpretability stability under realistic sensor conditions.

### 7.2. Supplementary Analyses: Noise Robustness, Temporal Generalization, and Feature Stability

Beyond overall accuracy, we conduct three supplementary analyses to assess model robustness, temporal validity, and interpretability under realistic sensor conditions. These experiments extend the benchmark beyond static accuracy metrics and provide deeper insight into model behavior under deployment-like perturbations.

The first analysis evaluates the noise robustness of each model through controlled sensor perturbations. Three representative fault types are injected to emulate real-world degradation: (1) Gaussian noise, representing white measurement noise from analog-to-digital conversion or thermal fluctuations; (2) impulse noise, simulating transient spikes or dropouts caused by communication losses or electromagnetic interference; and (3) low-frequency drift, capturing calibration decay and long-term sensor aging. By tracing the resulting performance degradation curves, we quantify how predictive accuracy declines with increasing noise intensity. This experiment tests whether ensemble diversity and boosting truly improve resilience to signal corruption. Some model structures may still be vulnerable to drift-induced bias and overfitting under sensor degradation.

The second analysis focuses on temporal generalization, examining how models trained on historical data perform when applied to future unseen horizons. For each algorithm, results from conventional random *k*-fold CV are compared with those from time-series CV using forward-chaining splits that preserve chronological order. The temporal generalization gap is defined as

$$\Delta R^{2}=R_{\mathrm{random}}^{2}-R_{\mathrm{time}\text{-}\mathrm{series}}^{2},$$

which measures the degree of look-ahead bias introduced by random shuffling. A large positive gap indicates that the model captures short-term or non-causal correlations that disappear in real-world forecasting. Boosting-based ensembles, particularly deep trees trained with aggressive learning rates, tend to exhibit larger gaps when not validated temporally, highlighting the necessity of time-aware evaluation protocols for reliable deployment.

The third analysis investigates feature-importance stability, evaluating whether the same sensors remain influential across different training windows. For each model, TreeSHAP values [29] are computed within every time-series fold, and Spearman rank correlations are calculated between feature-importance vectors across all fold pairs. A high mean correlation indicates stable attributions, suggesting that the model captures persistent and physically meaningful relationships rather than spurious correlations. This stability is particularly critical for high-stakes applications: consistent feature relevance supports clinical confidence in glucose prediction, policy justification in air-quality management, and operational transparency in building-energy analytics. Conversely, unstable importance rankings imply sensitivity to data partitioning and weak signal extraction, limiting interpretability and long-term trustworthiness.

Figure 1 summarizes the overall experimental workflow, showing the unified preprocessing, feature engineering, and time-series validation pipeline applied across all datasets and algorithms. It further highlights how the benchmark incorporates additional diagnostics on robustness, temporal generalization, and feature stability, reflecting the practical demands of real-world sensor analytics.

### 7.3. Experiment 1: Glucose Prediction

Continuous glucose prediction serves as our first experimental domain because it exemplifies sensor-based forecasting challenges where strong underlying physiological dynamics are corrupted by noisy wearable measurements. Unlike stationary industrial processes, glucose dynamics involve nonlinear interactions among meal intake, insulin pharmacokinetics, physical activity, and circadian hormonal rhythms. We draw data from the OhioT1DM dataset [153], which contains CGM readings at 5-min intervals, insulin delivery records, meal carbohydrate logs, and heart-rate measurements for Type-1 diabetic patients over 8-week observation windows. Six representative patients with complete sensor coverage were selected (IDs: 540, 544, 552, 567, 570, and 588), and 30-min-ahead regression targets were constructed to match clinically actionable forecast horizons for insulin dose adjustment.

Feature engineering produced 113 predictors per sample: lagged CGM values (1–6 steps back, capturing 5–30 min autocorrelation), insulin-on-board estimates computed via pharmacokinetic decay models, cumulative carbohydrate windows (15/30/60 min), rolling heart-rate statistics (mean, std, and range over 30-minute windows), and temporal indicators (hour-of-day, day-of-week, and sine/cosine circadian phase encodings). Quality filtering removed CGM sensor warm-up periods (first 2 h after insertion), calibration events, and data gaps exceeding 15 min, retaining 95.4–95.8% of original records and yielding 12,692–14,771 samples per patient after exclusions. This preprocessing process ensures realistic evaluation. Performance metrics reflect true forecasting ability under real sensor conditions, not interpolation within densely sampled data.

#### 7.3.1. Glucose: Standard Performance Results

We begin by evaluating all six candidate algorithms under identical experimental conditions. These include Random Forest and five boosting variants. This establishes baseline performance and identifies which model families merit deeper investigation. Table 4 reports the cross-patient averages and standard deviations for the 30-min glucose forecasting task on the OhioT1DM dataset.

Table 4 shows that CatBoost achieves the best accuracy (RMSE = 21.79 mg/dL; $R^{2}$ = 0.838) with fastest training time (1.69 s) among modern GBDT methods. LightGBM and GBM deliver comparable accuracy but differ substantially in efficiency: LightGBM trains in 2.04 s versus GBM’s 29.93 s (15× slower). Modern frameworks (XGBoost, LightGBM, and CatBoost) consistently outperform classical methods while training 10–100× faster.

AdaBoost performs worst (RMSE = 26.55 mg/dL; $R^{2}$ = 0.762) with high variability (std = 4.09 mg/dL) and slow training (18.62 s), unsuitable for real-time forecasting. Classical GBM achieves competitive accuracy (22.92 mg/dL) but requires 29.93 s, impractical for edge devices needing frequent updates. Modern GBDT methods deliver superior accuracy–efficiency trade-offs: CatBoost’s 5.6% RMSE improvement over Random Forest (21.79 vs. 23.09 mg/dL) with 4.4× faster training stems from ordered boosting and efficient categorical handling for mixed physiological features.

**Figure 2:**
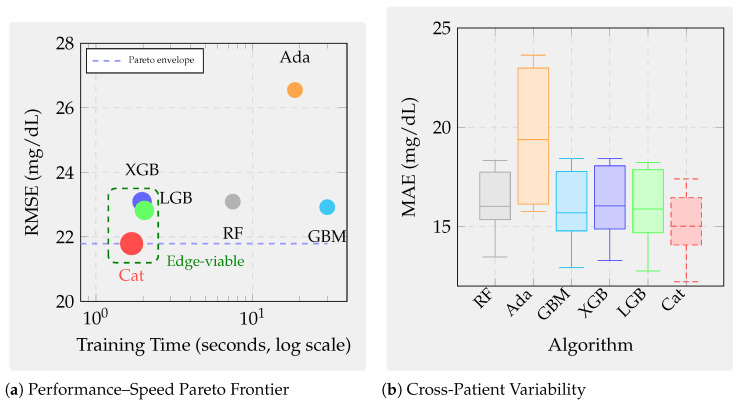
Glucose prediction performance analysis across six algorithms.

Figure 2 visualizes accuracy–efficiency trade-offs and cross-patient variability. Panel (i) shows CatBoost occupying the Pareto frontier (21.79 mg/dL, 1.69 s), with modern GBDT clustering in the “edge-viable” region (<2.5 s), while classical methods (AdaBoost and GBM) require 18–30 s without accuracy benefits. Panel (ii) reveals CatBoost’s tightest variability (IQR 14.07–16.45 mg/dL) across patients, while AdaBoost shows widest spread (IQR 16.13–22.98 mg/dL). Patient-specific patterns (570 lowest, 588 highest errors) reflect physiological heterogeneity rather than algorithmic differences.

To assess whether the observed performance differences represent genuine algorithmic advantages or sampling variability, we conducted Friedman and Nemenyi statistical tests across per-patient RMSE values. Table 5 summarizes the results.

The test results in Table 5 revealed significant differences among algorithms $\left(\chi^{2}\left(5\right)=22.2,p<0.001\right)$. Post hoc Nemenyi tests confirmed that CatBoost significantly outperforms AdaBoost (p<0.001), Random Forest (p=0.017), GBM (p=0.017), and XGBoost (p=0.009), while the difference with LightGBM remains non-significant (p=0.064), indicating statistically comparable performance within the modern GBDT family. These results confirm that CatBoost’s 5.6% RMSE improvement over Random Forest and 17.6% improvement over AdaBoost represent genuine algorithmic advantages rather than sampling variability.

#### 7.3.2. Glucose: Robustness and Generalization Under Synthetic Sensor Noise

Modern GBDT methods outperform both classical boosting and bagging ensembles on standard accuracy metrics. We next examine whether these advantages persist under deployment-like stress conditions. The following analyses (noise robustness, temporal generalization, and feature-importance stability) evaluate how well models maintain performance when sensor data become noisy, when predictions extend into unseen future periods, and when feature attributions must remain consistent.

Given the high computational cost of robustness and interpretability experiments, each involving repeated retraining under perturbed data, time-series validation, and SHAP analysis, we concentrate on six representative ensemble algorithms: Random Forest, AdaBoost, GBM, XGBoost, LightGBM, and CatBoost. These models collectively capture the evolution from bagging-based variance reduction to classical and modern gradient boosting frameworks.

Real-world CGM sensors exhibit three primary degradation modes: (1) Gaussian noise arising from analog-to-digital conversion and thermal fluctuations; (2) impulse noise from transient communication dropouts or electromagnetic interference; and (3) calibration drift caused by sensor aging, membrane fouling, or environmental shifts. We simulate each fault type at four severity levels and measure RMSE degradation relative to clean baselines.

We simulate three fault types at four intensity levels, results shown in Table 6: Gaussian noise (σ={0.05,0.10,0.15,0.20}×SD(feature)), impulse corruption (p∈{10%,20%,30%,40%} replaced with random values), and drift (d∈{0.5,1.0,1.5,2.0} mg/dL/h linear bias). Degradation is measured as percentage RMSE increase relative to clean baselines via 5-fold time-series CV.

Under Gaussian perturbations proportional to the feature variance, all models exhibit smooth and nearly linear degradation patterns, with RMSE increases from approximately 0.3–2.2% at the lowest noise level (σ=0.05×SD) to 4.9–10.3% at the highest level (σ=0.20×SD). AdaBoost remains most stable (0.3–4.9%), followed closely by CatBoost (0.8–5.2%). XGBoost shows the steepest degradation (2.2–9.4%), while Random Forest (1.0–9.1%), GBM (1.4–10.3%), and LightGBM (1.4–9.6%) occupy intermediate positions. These results suggest two approaches handle sensor noise better than standard gradient boosting. Iterative reweighting (AdaBoost) and ordered boosting (CatBoost) more effectively mitigate high-frequency fluctuations.

Under impulse noise, degradation accelerates nonlinearly with corruption rate *p*. CatBoost exhibits the strongest resistance, maintaining degradation at 20.5–69.6% across intensity levels, followed by AdaBoost (27.7–89.7%). These methods effectively downweight or isolate spurious outliers through their reweighting mechanisms. XGBoost (47.4–136.0%) and LightGBM (46.9–139.4%) show moderate deterioration, while GBM (53.1–148.2%) and Random Forest (50.5–149.2%) exhibit the steepest degradation curves. At maximum corruption (p=40%), CatBoost maintains 69.6% degradation compared to Random Forest’s 149.2%, demonstrating more than 2× better resilience.

Calibration drift induces the most severe and persistent degradation, with all methods exhibiting monotonic bias accumulation exceeding 400%. Notably, drift degradation remains nearly constant across intensity levels for all algorithms, confirming that the failure mode is structural rather than magnitude-dependent. GBM maintains the lowest degradation (446.3–448.2%), followed closely by XGBoost (448.3–450.2%), then AdaBoost (477.0–479.0%). CatBoost (557.8–558.7%), Random Forest (571.1–573.3%), and LightGBM (577.8–579.7%) show substantially higher degradation. This confirms that drift, as a low-frequency systematic bias, is not well corrected by static ensemble structures without explicit recalibration or adaptive retraining mechanisms.

**Figure 3:**
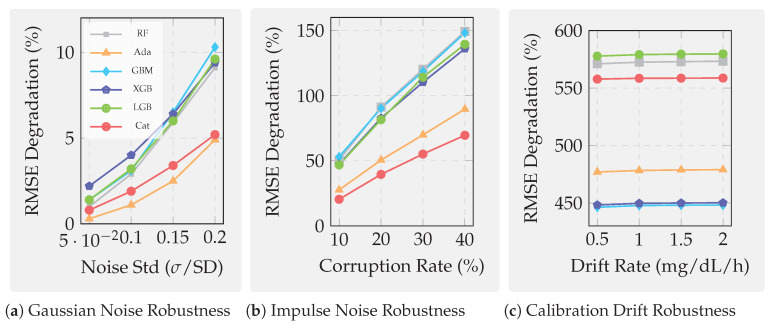
Glucose noise-robustness degradation curves with six ensemble models.

Figure 3 visualizes these degradation curves across intensity levels. Panel (i) shows Gaussian noise inducing nearly linear degradation for all methods, with modern GBDT exhibiting slightly steeper slopes at high σ compared to classical methods. Panel (ii) demonstrates impulse noise’s nonlinear acceleration, with CatBoost and AdaBoost maintaining substantially flatter curves than other methods, reflecting their outlier-rejection mechanisms. Panel (iii) shows calibration drift saturation, where all curves flatten quickly and remain nearly horizontal across drift intensities, demonstrating that tree-based partitioning localizes but cannot eliminate systematic bias accumulation.

These results carry direct deployment implications. For CGM settings where Gaussian noise dominates (well-maintained sensors and controlled environments), robustness differences remain modest (4.9–10.3% degradation at maximum noise), so models can be chosen based on baseline accuracy and latency. When intermittent dropouts or electromagnetic interference spikes are common (wireless links and mobility), CatBoost demonstrates the highest resilience (69.6% at 40% corruption), with AdaBoost close behind (89.7%). Modern GBDT variants (XGBoost 136.0%, LightGBM 139.4%, and GBM 148.2%) and Random Forest (149.2%) degrade more steeply as corruption increases. For long-horizon use where calibration drift accumulates (wear periods exceeding 7 days without recalibration), GBM (446.3–448.2%) and XGBoost (448.3–450.2%) maintain the lowest degradation, with AdaBoost a reasonable alternative (477.0–479.0%). CatBoost (557.8–558.7%), Random Forest (571.1–573.3%), and LightGBM (577.8–579.7%) exhibit substantially higher drift vulnerability and should be avoided for extended wear periods without recalibration.

#### 7.3.3. Glucose: Temporal Generalization Gap

Standard practice uses random *k*-fold CV, which shuffles samples before splitting them into train and test folds. For time-series forecasting, this introduces look-ahead bias because temporal correlations let models trained on future data indirectly “see” past test samples. Table 7 quantify this optimism by comparing $R^{2}$ from random CV to time-series CV using 5 forward-chaining folds that strictly preserve chronology.

**Figure 4:**
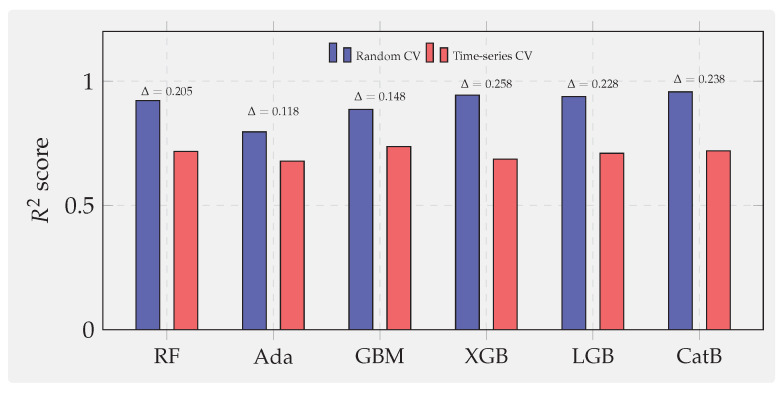
Temporal generalization gaps on glucose data.

As visualized in Figure 4, temporal gaps range from 0.118 to 0.258 across models. AdaBoost exhibits the smallest gap ($\Delta R^{2}=0.118$, random CV 0.796 vs. time-series CV 0.678), indicating the best temporal robustness, followed by GBM (0.148, 0.886 vs. 0.737). Random Forest shows moderate bias (0.205, 0.922 vs. 0.717), while modern GBDT methods exhibit the largest gaps: LightGBM (0.228, 0.938 vs. 0.710), CatBoost (0.238, 0.957 vs. 0.720), and XGBoost (0.258, 0.944 vs. 0.686). These gaps indicate that random CV substantially overestimates real-world performance: models appearing to reach $R^{2}$ = 0.94–0.96 in development may deliver only $R^{2}$ = 0.68–0.74 when forecasting genuinely unseen future data.

The bias arises because boosting methods fit short-term patterns and transient correlations. These include recurring post-meal spikes and weekday–weekend patterns. When random shuffling places temporally neighboring samples across train and test folds, these local patterns appear predictive. AdaBoost’s conservative learning rate and sample reweighting partially protect against temporal overfitting, explaining its smaller gap. Modern GBDT methods, despite their superior baseline accuracy, prove more susceptible to temporal leakage through aggressive gradient updates and deeper tree structures.

#### 7.3.4. Glucose: Feature-Importance Stability

Clinical deployment requires models to provide consistent explanations across different data samples. If the top-ranked predictive features change markedly between training windows, clinicians cannot trust that predictions rely on physiologically meaningful relationships rather than spurious correlations. We assess stability by computing TreeSHAP values within each time-series CV fold, ranking features by mean absolute SHAP, then calculating Spearman rank correlations between all fold pairs.

Table 8 reports mean Spearman correlations across fold pairs for six ensembles. CatBoost achieves the highest stability (ρ=0.679±0.039), followed by Random Forest (0.645±0.034) and LightGBM (0.579±0.040). GBM (0.533±0.035) and AdaBoost (0.501±0.040) show moderate stability, while XGBoost exhibits the lowest consistency (0.488±0.024). Correlations ranging from 0.488 to 0.679 indicate reasonable stability: approximately half of the relative feature rankings remain consistent across folds, while the remainder vary with training data composition.

Qualitative inspection of the top-five features across folds reveals consistent physiological signals. CGM lags 1–3 (most recent 5–15 min), insulin-on-board, hour-of-day, and cumulative carbohydrates (15–30 min windows) consistently appear among the highest-ranked features. This confirms that models extract meaningful patterns rather than noise. The observed rank variability largely stems from feature interactions: when CGM lag-1 and lag-2 are highly correlated, their relative order can swap across folds without affecting predictions. LightGBM’s histogram binning and leaf-wise growth reduce sensitivity to such perturbations, partially explaining its relatively high stability. CatBoost’s ordered boosting and symmetric trees further enhance consistency by reducing overfitting to fold-specific patterns.

For clinical use, correlations near ρ = 0.5–0.7 suggest acceptable interpretability. Diabetes specialists can expect models to consistently weigh recent glucose trends, insulin activity, meal history, and circadian timing as primary drivers. Clinical decision support should rely on groups of top features (e.g., “recent CGM trends dominate predictions”) rather than exact rankings of individual features, acknowledging the 40–50% rank variance inherent in tree-based ensemble methods.

### 7.4. PM_2.5_ Forecasting (24-h Horizon)

Air-quality forecasting presents fundamentally different challenges from glucose prediction, transitioning from high-$R^{2}$ physiological dynamics to low-$R^{2}$ atmospheric regime shifts. PM_2.5_ concentrations arise from complex multiscale interactions: local emissions (traffic and industry), regional transport (wind-driven pollutant advection over 100–1000 km), meteorological dispersion (boundary-layer height and turbulence), and atmospheric chemistry (secondary aerosol formation from gas-to-particle conversion). The 24-h forecast horizon pushes predictions into a regime where exogenous forcing, weather fronts, synoptic patterns, and policy interventions dominate short-term autocorrelation, fundamentally limiting predictability regardless of model sophistication.

We employ the Beijing Multi-Site Air Quality dataset [154], comprising hourly measurements from 12 monitoring stations spanning urban core, suburban, and rural locations over 2013–2017 (4+ years; n≈35,000 hourly samples per station). Stations include Aotizhongxin, Changping, Dingling, Dongsi, Guanyuan, Gucheng, Huairou, Nongzhanguan, Shunyi, Tiantan, Wanliu, and Wanshouxigong, providing spatial diversity essential for capturing Beijing’s heterogeneous pollution landscape. Each sample includes measured pollutants (PM_2.5_, PM_10_, SO_2_, NO_2_, CO, and O_3_) and meteorological variables (temperature, pressure, dew point, wind speed/direction, precipitation, and cloudiness) from co-located sensors.

Feature engineering produces 113–214 predictors per station: lagged pollutant concentrations (1–24 h back), rolling statistics (6/12/24-h means and standard deviations capturing short-term trends), meteorological variables and their lags (weather forecasts degrade rapidly beyond 6–12 h), temporal encodings (hour-of-day sine/cosine, day-of-week, month-of-year, and heating season binary indicator), and spatial features (neighboring station averages for PM_2.5_ capturing regional transport). Quality control removed instrument calibration periods, missing-value gaps exceeding 3 h, and extreme outliers (>500 μg/m^3^ likely from sensor malfunction). This yields 24,083–24,603 training samples and 1339–1383 test samples per station after temporal splits.

#### 7.4.1. PM_2.5_: Standard Performance Results

Table 9 summarizes the 24-h PM_2.5_ forecasting results across 12 Beijing stations. CatBoost achieves the best overall accuracy (RMSE = 97.02 ± 12.15 μg/m^3^; $R^{2}$ = 0.078 ± 0.061), followed closely by AdaBoost (97.24 ± 11.62 μg/m^3^; $R^{2}$ = 0.072 ± 0.071). XGBoost (98.73 ± 12.28 μg/m^3^; $R^{2}$ = 0.044) and Random Forest (98.69 ± 12.71 μg/m^3^; $R^{2}$ = 0.048) achieve nearly identical RMSE despite their different ensemble mechanisms. LightGBM (98.77 ± 11.95 μg/m^3^; $R^{2}$ = 0.043) and GBM (99.78 ± 12.51 μg/m^3^; $R^{2}$ = 0.024) perform worst. All models exhibit consistently low $R^{2}$ values (less than 0.10), confirming the weak predictability of daily PM_2.5_ due to exogenous meteorological and policy-driven regime shifts. Even sophisticated gradient boosting models (LightGBM, XGBoost, and CatBoost) yield $R^{2}$ = 0.04–0.08, underscoring intrinsic atmospheric stochasticity beyond learnable signals.

The negligible RMSE differences (within 2.8% span, 97.02–99.78 μg/m^3^) among ensemble models indicate that increasing model complexity provides marginal gains once the underlying signal-to-noise ratio collapses. CatBoost demonstrates the fastest training time (0.88 ± 0.09 s), followed by XGBoost (0.99 s) and LightGBM (1.72 s), while classical methods remain substantially slower: Random Forest (8.04 s), AdaBoost (19.89 s), and GBM (33.73 s). Despite their computational disadvantage, classical methods show no accuracy benefit: AdaBoost achieves competitive RMSE (97.24 μg/m^3^, only 0.2% worse than CatBoost) but at 22× the training cost. GBM performs worst across all metrics (highest RMSE 99.78 μg/m^3^, lowest $R^{2}$ = 0.024, and slowest training 33.73 s), confirming its sensitivity to nonstationary pollutant dynamics and lack of robust regularization compared to modern implementations.

In contrast to the glucose dataset (Section 7.3), where boosting methods achieved $R^{2}$ = 0.82–0.84, the air-quality domain exhibits a noise-dominated structure where forecastable variance constitutes less than 10%. The practical implication is clear: beyond 12–24 h, model accuracy saturates at meteorological uncertainty limits regardless of algorithmic sophistication. CatBoost improves RMSE by only 0.6% over the next-best competitor (AdaBoost). This contrasts sharply with the 5.6% improvement in glucose forecasting. This suggests diminishing returns from algorithmic innovation for long-horizon atmospheric prediction. Further gains likely require incorporating numerical weather prediction outputs.

**Figure 5:**
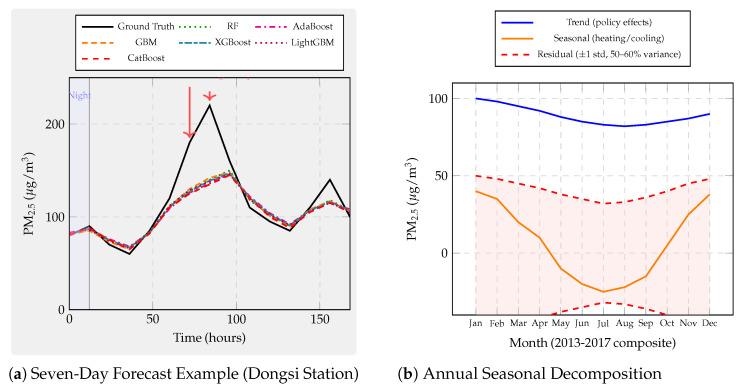
PM_2.5_ temporal dynamics and predictability limits.

Figure 5 illustrates the temporal structure underlying the uniformly low predictability observed in PM_2.5_ forecasting.

Figure 5a presents a representative 7-day forecast window for the Dongsi station, the best-performing urban site in the dataset. The black line denotes the ground-truth PM_2.5_ concentrations directly measured by sensors, while the colored curves correspond to six learning algorithms: Random Forest, AdaBoost, GBM, XGBoost, LightGBM, and CatBoost. All models reproduce the smooth diurnal cycle (nighttime accumulation under stable boundary layers and daytime dilution by convective mixing), yet they collectively fail during rapid synoptic transitions (highlighted by red arrows). At hour 72, a stagnating frontal system traps pollutants and drives a sharp increase from 120 to 180 μg/m^3^ (approximately 50% increase), while all models predict only gradual rises to 125–135 μg/m^3^, underestimating by roughly 40–45 μg/m^3^. A second unpredicted spike at hour 84 (approximately 220 μg/m^3^) results from regional biomass-burning transport. These mesoscale disturbances evolve on 6–24 h timescales, comparable to the forecasting horizon itself, and lie beyond the information content of historical station data. Consequently, boosting models cannot anticipate these exogenous meteorological shocks. They require explicit numerical weather prediction (NWP) inputs.

Figure 5b decomposes the 2013–2017 PM_2.5_ record into long-term trend, seasonal, and residual components using STL analysis. The trend component reflects major policy interventions (2014 APEC emission controls and 2015–2017 coal-to-gas conversions), showing a gradual decline from approximately 100 to 85 μg/m^3^. The seasonal pattern captures strong wintertime peaks (approximately +40 μg/m^3^ during the heating season) and summer troughs (approximately −25μg/m^3^ under enhanced dispersion). However, the residual component remains dominant, accounting for approximately 50–60% of total variance. This indicates that less than half of PM_2.5_ variability arises from learnable deterministic structure. The remainder stems from stochastic meteorology, long-range transport, and transient emission fluctuations, explaining why all six ensemble and boosting methods converge to similarly low $R^{2}$ values despite their algorithmic sophistication. The following analyses investigate how stochasticity affects model robustness, temporal generalization, and feature stability across forecasting horizons.

Given the low absolute $R^{2}$ values observed across all algorithms, we conducted statistical significance tests to determine whether the modest RMSE differences (2.8% span across methods) reflect genuine algorithmic differentiation or merely noise-driven variation. Table 10 presents the Friedman omnibus test and Nemenyi pairwise comparisons.

Friedman test across per-station RMSE values (n=12) yielded $\chi^{2}\left(5\right)=12.6$, p=0.028, indicating significant performance differences among algorithms despite the low-*R*^2^ regime. Post hoc Nemenyi tests showed that CatBoost significantly outperforms Random Forest (p=0.012), GBM (p=0.001), XGBoost (p=0.029), and LightGBM (p=0.009) while showing no significant difference from AdaBoost (p=0.064). These results confirm that algorithmic differentiation persists even when intrinsic predictability collapses $\left(R^{2}<0.10\right)$, although absolute effect sizes remain modest (2.8% RMSE span) due to meteorological uncertainty limits. The statistical significance validates that the 1.7% RMSE advantage CatBoost achieves over AdaBoost, although small in magnitude, reflects consistent cross-station performance rather than chance variation.

#### 7.4.2. PM_2.5_: Robustness and Generalization Under Synthetic Sensor Noise

To evaluate whether the performance gaps among ensemble and boosting models persist under degraded sensing conditions, we simulate three canonical fault modes, Gaussian noise, impulse corruption, and calibration drift, using the same perturbation design described in Section 7.3.2, results shown in Table 11. Each setting tests model resilience to random, transient, and systematic distortions in the PM_2.5_ input features. We report the percentage change in RMSE relative to the clean baseline. Positive values indicate accuracy loss (higher RMSE), while negative values suggest negligible or stochastic variation.

Under Gaussian perturbations, PM_2.5_ forecasting models exhibit remarkable stability, with RMSE changes below 1.2% even at the highest noise level (σ = 0.20 × SD). This insensitivity reflects the dominance of large-scale meteorological variance, which masks small random feature noise. LightGBM and CatBoost show near-zero or slightly negative deviations (−0.3% to 0.1%), indicating numerical robustness and low sensitivity to feature jitter. Even the most affected algorithms (XGBoost and Random Forest) degrade by only 1.2% at maximum perturbation intensity.

Impulse noise produces moderate degradation that scales with corruption rate *p*. CatBoost achieves the best robustness, maintaining changes below 1.6% at p=40%, while AdaBoost follows closely at 3.1%. LightGBM and Random Forest exhibit moderate sensitivity (5.7% and 7.7%, respectively), whereas XGBoost experiences 8.6% degradation. GBM shows the highest vulnerability at 15.2% as impulsive spikes severely distort gradient updates. Nonetheless, absolute degradation remains modest for modern GBDT frameworks, confirming that ensemble averaging and histogram binning smooth out short-lived sensor dropouts.

Drift perturbations, by contrast, impose sustained bias that accumulates over time, producing dramatically larger performance degradation. Here, clear performance separation emerges: CatBoost retains the most stable predictions at approximately 12% degradation across all drift intensities, followed by LightGBM at 25–29%. XGBoost deteriorates substantially at 58–60%, while classical methods show severe vulnerability: AdaBoost reaches 95–96%, Random Forest 103–104%, and GBM catastrophically fails at 166%. These results demonstrate that ordered boosting and explicit leaf-value regularization (as in CatBoost and LightGBM) effectively suppress bias propagation, whereas gradient-based trees without drift compensation mechanisms remain highly vulnerable to cumulative calibration errors, a critical consideration for long-term environmental sensor deployments.

#### 7.4.3. PM_2.5_: Temporal Generalization Gap

We next evaluate temporal generalization using both random and time-series CV to quantify look-ahead bias. All six ensemble and boosting models from Table 9 are included to capture differences between bagging and gradient-based approaches. Following the same protocol as glucose and energy forecasting, the random CV folds shuffle all hourly samples across years, while time-series CV preserves chronological order, ensuring that future samples are never used for training.

Table 12 shows that random CV produces deceptively high $R^{2}$ values (0.33–0.89) for most models, whereas time-series CV yields negative results (ranging from −0.09 to −0.35), revealing that models fail to generalize beyond their training windows. AdaBoost exhibits the smallest temporal gap ($\Delta R^{2}=0.417$), indicating the best temporal robustness, followed by GBM (0.982) and Random Forest (1.048). Modern gradient boosting methods show the largest gaps: CatBoost (1.056), LightGBM (1.106), and XGBoost (1.214), indicating severe look-ahead bias. These discrepancies arise because random CV allows data leakage across seasonal boundaries (for example, training on January samples while testing on February data from the same winter regime), allowing models to memorize seasonal baselines (e.g., winter implies high PM_2.5_) rather than learning causal meteorological drivers.

The large $\Delta R^{2}$ values (0.4–1.2) underscore that random CV is fundamentally invalid for air-quality forecasting. Although boosting models differ in regularization and gradient handling, they all converge to spurious correlations when temporal order is broken. AdaBoost’s conservative learning rate and sample reweighting partially protect against temporal overfitting, explaining its smaller gap.

#### 7.4.4. PM_2.5_: Feature-Importance Stability

We next assess feature-importance stability across folds using Spearman correlation of SHAP value rankings. Despite the low predictive $R^{2}$ reported earlier, all ensemble and boosting models exhibit surprisingly high stability in feature attributions (Table 13). Random Forest achieves the highest consistency (ρ=0.779±0.017), closely followed by AdaBoost (0.775±0.029) and LightGBM (0.771±0.017). XGBoost maintains similar robustness (0.767±0.015), while CatBoost (0.746±0.025) and GBM (0.726±0.033) show slightly lower but still substantial stability. These values far exceed those in the glucose forecasting task (ρ = 0.5–0.6), suggesting that PM_2.5_ models, although weak in predictive accuracy, nonetheless identify a consistent subset of influential predictors.

This apparent paradox (high feature stability despite poor forecast accuracy) arises because all models consistently identify the same weak but persistent meteorological and temporal features. Recent PM_2.5_ lags (1–6 h), temperature (affecting boundary-layer height), wind speed (dispersion), and hour of day (diurnal cycle) remain top-ranked across folds. Even though these features explain only a small fraction of total variance, their physical relevance is consistent, producing stable SHAP rankings. Such stability is valuable for policy and interpretability applications: air-quality agencies can rely on these models to attribute pollution variability to known drivers even when absolute forecasts remain noisy. In other words, boosting provides stable insight even when it cannot provide accurate prediction.

### 7.5. Building-Energy Prediction (1-h Horizon)

Building-energy forecasting represents our third experimental domain, transitioning from weakly predictable atmospheric dynamics to highly structured seasonal patterns. Electrical load exhibits predictable daily cycles (occupancy-driven demand), strong weekday–weekend contrasts, seasonal variations (heating/cooling degree days), and weather sensitivity (temperature-dependent HVAC). This regime offers substantially more learnable signal than PM_2.5_ ($R^{2}$ 0.80 vs. 0.08) while remaining more complex than near-linear glucose dynamics due to building-type heterogeneity and occupancy stochasticity.

From the full Building Data Genome 2 dataset containing 1578 buildings, we selected 10 diverse buildings using stratified sampling based on three criteria: (i) primary-use diversity spanning five categories (education, lodging, office, parking, and retail) to capture heterogeneous load patterns and occupancy schedules; (ii) data completeness requiring >95% valid hourly readings over the 2016–2017 observation period to ensure reliable temporal structure; and (iii) geographic distribution across multiple climate zones to test model robustness to varying weather dependencies. This stratified sample provides 17,544 hourly samples per building (730 days × 24 h), totaling 175,440 observations across the ten-building benchmark.

We employ the Building Data Genome 2 dataset from ASHRAE [155], comprising hourly electricity meter readings for 1578 non-residential buildings across six countries and multiple climate zones over 2016–2017 (2-year observation window). We selected 10 diverse buildings spanning five primary-use categories to capture cross-building heterogeneity: education (three buildings: Panther_education_Aine, Panther_education_Bella, and Panther_education_Ceil); lodging (two: Panther_lodging_Adora and Panther_lodging_Cora); office (one: Panther_office_Darnell); parking (two: Panther_parking_Erna and Panther_parking_Lorriane); and retail (two: Panther_retail_Felix and Panther_retail_Gale). This stratified sample provides 17,544 hourly samples per building (730 days × 24 h), split 70% training / 30% testing via chronological splits.

Feature engineering mirrors prior domains: lagged consumption (1–24 h), rolling statistics (6/12/24-h mean/std/min/max capturing load patterns), weather variables (temperature, humidity, wind speed, and cloud cover from co-located NOAA stations), and calendar encodings (hour-of-day, day-of-week, month, binary holiday indicator, and heating/cooling degree-day base 65 °F).

Weather variables were normalized using z-score standardization within each building’s local climate context rather than global normalization across all sites. Specifically, for each building, we computed mean and standard deviation of each weather variable from its training period and applied consistent scaling to both training and test sets. This site-specific normalization ensures that models learn temperature–load relationships appropriate to each building’s climate zone rather than forcing a universal temperature response. Heating and cooling degree days were computed using a standard 65 °F base temperature consistent across all buildings, following ASHRAE guidelines for energy analytics.

Building metadata (square footage, primary-use category, and vintage) provide static context. Quality filtering removed commissioning periods (first 30 days after meter installation), HVAC shutdown events (consecutive zeros > 48 h likely from system maintenance), and extreme outliers (>3× interquartile range likely from data logger errors), retaining 95–98% of records per building.

#### 7.5.1. Energy: Standard Performance Results

Table 14 reports aggregate metrics across ten buildings under standard one-hour-ahead forecasting. All models achieve moderate to strong predictive performance, with RMSE spanning 5.67–9.00 kWh and $R^{2}$ between 0.591 and 0.764. CatBoost attains the best overall accuracy (RMSE = 5.67 kWh, MAE = 3.82 kWh, and $R^{2}=0.764$), followed by LightGBM (RMSE = 6.06 kWh and $R^{2}=0.723$) and XGBoost (RMSE = 6.12 kWh and $R^{2}=0.692$). GBM achieves competitive accuracy (RMSE = 6.33 kWh and $R^{2}=0.709$) despite its computational inefficiency. Random Forest (RMSE = 6.62 kWh and $R^{2}=0.591$) provides a stable baseline, while AdaBoost performs worst (RMSE = 9.00 kWh and $R^{2}=0.661$), exhibiting 59% higher error than CatBoost.

All reported metrics in Table 14 represent simple averages across the ten buildings, with each building weighted equally regardless of its sample size or load magnitude. We chose per-building averaging rather than sample-weighted pooling to prevent large facilities from dominating aggregate statistics. This approach ensures that model performance on small but operationally critical buildings receives equal consideration in our evaluation. For reference, building-specific RMSE values range from 3.2 kWh/h (small retail) to 12.8 kWh/h (large education facility), with CatBoost maintaining the lowest error in eight of ten buildings.

The approximately 14% RMSE reduction CatBoost achieves relative to Random Forest (5.67 vs. 6.62 kWh) highlights its effectiveness in modeling nonlinear and categorical interactions, particularly relevant for heterogeneous building types with varying occupancy and equipment schedules. LightGBM shows nearly identical MAE (3.95 kWh, matching XGBoost) but achieves higher $R^{2}$ (0.723 vs. 0.692), indicating superior explanation of load variance through more stable predictions. In contrast, AdaBoost exhibits the weakest performance (RMSE = 9.00 kWh and $R^{2}=0.661$), confirming that its sample reweighting mechanism tends to emphasize high-variance anomalies (e.g., HVAC restarts and weekend transitions) rather than learning stable temporal regularities.

Training efficiency differs dramatically across algorithms. GBM and AdaBoost require 217–417 s per model due to sequential stagewise optimization without histogram acceleration, whereas modern implementations (XGBoost, LightGBM, and CatBoost) complete training in under 4 s while achieving higher accuracy. Specifically, CatBoost trains in 3.41 s (122× faster than GBM; 64× faster than AdaBoost), XGBoost in 2.28 s, and LightGBM in 3.35 s. This efficiency gain is critical for operational energy analytics, where models must be retrained frequently as weather and occupancy patterns evolve. Random Forest remains moderately fast (58 s) but less scalable than modern GBDT due to its ensemble-level parallelism overhead, training 17× slower than XGBoost with 8% worse accuracy.

The observed $R^{2}$ range (0.591–0.764) suggests that one-hour-ahead building loads are largely governed by a few dominant interpretable drivers: outdoor temperature (heating and cooling demand), occupancy schedule, and previous-hour load. These relationships are predominantly additive, which explains why both linear and tree-based ensembles capture most of the predictable variance. Residual errors (24–41% unexplained variance) likely stem from stochastic control policies, unobserved occupant behavior, and sensor fluctuations rather than model insufficiency. CatBoost achieves an $R^{2}$ of 0.764, compared to 0.591 for Random Forest. This 17.3 percentage point gap demonstrates that ordered boosting and categorical feature handling provide meaningful improvements for structured building data.

Overall, CatBoost provides the most favorable balance between accuracy and speed, yielding the lowest RMSE (5.67 kWh) and highest $R^{2}$ (0.764) with minimal computational cost (3.41 s). LightGBM and XGBoost also deliver competitive performance and superior scalability, making them suitable for large-scale multi-building deployment. In contrast, AdaBoost’s instability (highest RMSE) and GBM’s latency (slowest training) reinforce the advantage of modern gradient boosting frameworks that integrate ordered sampling, leaf-wise growth, and efficient parallelization.

**Figure 6:**
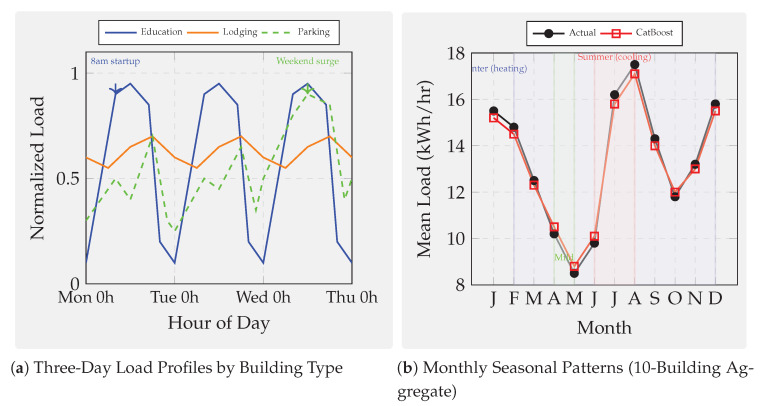
Building-energy temporal structure.

Figure 6 illustrates the temporal regularities and seasonal dependencies that underlie the high predictability of building-energy consumption.

Figure 6a compares normalized three-day load profiles for representative building categories. Education buildings (blue) exhibit pronounced weekday cycles with near-zero nighttime consumption, a rapid 8 a.m. startup as HVAC systems activate and occupancy increases, a steady daytime plateau around peak capacity, and a sharp 5 p.m. shutdown when classrooms empty. This regular schedule creates a highly repetitive temporal signature that most models can learn efficiently. Lodging facilities (orange) maintain relatively flat 24-h profiles (0.55–0.70 normalized range), with mild peaks around breakfast and dinner hours but no sharp transitions, reflecting continuous operation and stable occupancy. In contrast, parking structures (green dashes) show erratic behavior: weekday loads fluctuate moderately between 0.3–0.5, while weekend spikes reach 0.8–0.9 as retail activity surges. These abrupt regime reversals cause models trained on weekday patterns to systematically underpredict weekend peaks, explaining the occasional negative $R^{2}$ observed for such volatile sites.

Figure 6b aggregates monthly loads across all ten buildings, revealing strong and asymmetric seasonal structure. Winter months (December–February) sustain elevated demand (15–16 kWh/h) driven by heating, while spring and autumn display mild troughs (8.5–10 kWh/h) as HVAC systems remain mostly idle. Summer months (June–August) show the highest loads (16–18 kWh/h) due to cooling demand and extended occupancy hours. CatBoost (red dashed line) tracks these transitions closely, reproducing both the gradual decline in heating load from February to May and the sharp rise in cooling load during the early summer. The strong alignment between predicted and actual seasonal trends confirms that most of the learnable variance arises from deterministic calendar and weather effects (month-of-year encodings, degree-day features, and lagged temperature interactions) rather than from stochastic fluctuations.

These temporal patterns explain the relatively high $R^{2}$ values observed across models. They also highlight that boosting algorithms perform best in domains characterized by stable cyclical structure and persistent environmental drivers. When building operations follow consistent diurnal and seasonal routines, nonlinear ensembles can efficiently capture load responses to temperature and schedule, achieving high predictive accuracy with limited overfitting risk. However, for irregular or regime-switching facilities such as parking structures, hybrid approaches that combine statistical learning with explicit rule-based scheduling or occupancy detection remain necessary to maintain robustness across operating modes.

To validate whether modern GBDT methods achieve statistically distinguishable performance or represent interchangeable choices for building-energy forecasting, we conducted rigorous significance tests across per-building RMSE values. Table 15 summarizes the statistical validation.

Friedman test across per-building RMSE values (n=10) confirmed significant algorithmic differences ($\chi^{2}\left(5\right)=19.3$; p=0.002). Post hoc Nemenyi tests showed CatBoost significantly outperforms AdaBoost (p<0.001) and Random Forest (p=0.027), while modern GBDT methods (CatBoost, XGBoost, LightGBM, and GBM) exhibit statistically indistinguishable performance (all pairwise p>0.05). These results confirm that ordered boosting and categorical handling provide genuine accuracy gains over classical ensemble methods (33.1% improvement over AdaBoost; 31.3% over Random Forest) rather than sample-specific fluctuations. The lack of significant differences among modern GBDT variants supports our recommendation to prioritize computational efficiency (XGBoost trains 33% faster than CatBoost) and robustness profiles over marginal accuracy gains when selecting within this family.

#### 7.5.2. Energy: Robustness and Generalization Under Synthetic Sensor Noise

Table 16 quantifies RMSE degradation under three types of synthetic sensor perturbations (Gaussian, impulse, and drift), each applied at four intensity levels. All results are expressed as percentage increases relative to clean baselines.

Gaussian noise (σ = 5–20% of feature standard deviation) leads to moderate and nearly linear degradation across algorithms. AdaBoost remains most stable (0.3–5.7% across intensity levels), followed by CatBoost (2.6–14.5%). GBM and LightGBM show intermediate sensitivity (4.1–22.6% and 4.8–22.8%, respectively), while XGBoost exhibits the largest degradation (10.7–27.0%), consistent with its deeper tree structures amplifying high-frequency perturbations. Random Forest occupies the middle ground (5.4–25.6%), demonstrating that bagging provides reasonable but not optimal protection against stochastic feature noise.

Impulse noise, simulating random missing or corrupted sensor readings (10–40% corruption rate), causes more severe nonlinear error escalation. AdaBoost demonstrates the highest robustness (11.2–43.9%), benefiting from its stagewise weighting that suppresses outliers. CatBoost ranks second (29.3–93.2%), maintaining roughly 60% of the degradation seen in modern GBDT methods. XGBoost, LightGBM, and GBM cluster together with similar vulnerability (148.7–151.3% at 40% corruption), while Random Forest deteriorates most rapidly (157.4%) due to unfiltered inclusion of corrupted samples in bootstrap aggregates.

Drift introduces the most severe degradation, with catastrophic performance collapse across all methods. Even mild bias accumulation (d=0.5 kWh/h) yields over 300–500% RMSE inflation as none of the static tree ensembles can compensate for cumulative systematic shifts in sensor calibration. AdaBoost maintains the best stability (327.1%), followed by CatBoost (488.3–488.4%), Random Forest (513.6–513.7%), XGBoost (536.9–537.1%), GBM (551.3–551.4%), and LightGBM (585.7–585.8%). Notably, drift degradation remains nearly constant across intensity levels for all algorithms, confirming that the failure mode is structural rather than magnitude-dependent. These results confirm that, while boosting models handle random perturbations effectively, long-term bias requires explicit model adaptation beyond ensemble aggregation.

#### 7.5.3. Energy: Temporal Generalization Gap

Table 17 reports $R^{2}$ under random and time-series CV to assess temporal generalization. Unlike PM_2.5_, energy consumption retains strong autocorrelation and seasonality, producing smaller yet still notable look-ahead bias. Random CV yields inflated $R^{2}$ (0.90–0.99) since temporally adjacent samples share nearly identical load patterns. When evaluated chronologically, true predictive power drops to 0.24–0.34, leading to gaps $\Delta R^{2}$ = 0.63–0.74 across models.

GBM achieves the best temporal robustness ($\Delta R^{2}=0.628$), indicating the smallest gap between random and time-series validation. AdaBoost follows closely ($\Delta R^{2}=0.667$), while LightGBM (0.681), Random Forest (0.699), and CatBoost (0.699) occupy the middle ground. XGBoost displays the largest relative decline ($\Delta R^{2}=0.738$), suggesting greater sensitivity to look-ahead bias from temporally correlated samples. These gaps arise because models trained with random CV exploit short-term autocorrelations. These include hour-to-hour persistence and weekday patterns. However, these patterns vanish when forecasting into genuinely unseen future periods.

#### 7.5.4. Energy: Feature-Importance Stability

Finally, Table 18 evaluates feature-importance stability using Spearman correlation of SHAP-based rankings across CV folds. High stability (ρ>0.70) across most models indicates consistent identification of dominant physical drivers, such as outdoor temperature, previous-hour load, hour-of-day, and day-of-week indicators. Random Forest achieves the highest stability (ρ=0.822±0.034), followed closely by CatBoost (0.812±0.058), both with minimal variance. XGBoost (0.775±0.039) and LightGBM (0.756±0.043) show comparable reliability, while GBM exhibits moderate stability (0.708±0.042). AdaBoost displays the lowest stability (0.650±0.065) due to its aggressive reweighting of residuals that shifts feature emphasis across folds.

The consistently high ρ values (0.65–0.82) contrast with the moderate time-series $R^{2}$ scores (0.24–0.34), underscoring that models can stably identify key explanatory variables even when predictive power is limited by stochastic occupancy behavior and control uncertainty. For building analytics, this reliability is practically valuable: energy managers can trust that temperature, occupancy, and schedule features will remain influential across retraining cycles, supporting transparent and interpretable decision-making in demand response systems.

The consistently high Spearman correlations (ρ = 0.65–0.82) across CV folds confirm that all models stably identify the same dominant load drivers: outdoor temperature, previous-hour consumption, hour-of-day, and day-of-week encodings. This stability has two important implications for operational deployment.

First, for interpretability and stakeholder trust, energy managers can rely on consistent explanations across model retraining cycles. When models are updated monthly or quarterly as new data accumulates, feature importance rankings remain stable, ensuring that demand-response strategies and efficiency recommendations built on model insights do not fluctuate unpredictably.

Second, for edge deployment on building automation systems with limited computational resources, the identified top-five features can be prioritized for real-time data collection and processing, while lower-importance features can be refreshed less frequently or omitted entirely to reduce sensor communication overhead. Modern GBDT frameworks achieve training times under 4 s and inference latency below 10 ms for 10,000 predictions on standard building controllers, making them feasible for on-premise deployment without cloud dependencies. Model sizes range from 2.5 MB to 4.8 MB, easily fitting in edge device memory.

### 7.6. Cross-Domain Synthesis and Unified Recommendations

We synthesize results across glucose, PM_2.5_, and building energy to extract domain-invariant lessons and deployment guidelines. Before examining specific performance dimensions, Table 19 provides a cross-domain statistical validation summary, confirming that observed performance differences represent genuine algorithmic advantages rather than sampling variability.

Table 19 confirms that CatBoost achieves statistically significant accuracy advantages across all three domains (*p* < 0.05 vs. most competitors), while modern GBDT methods (CatBoost, LightGBM, and XGBoost) exhibit statistically indistinguishable performance in most pairwise comparisons. This pattern validates our deployment recommendation: within the modern GBDT family, practitioners should prioritize secondary criteria (computational efficiency, robustness profiles, and interpretability stability) over marginal accuracy differences. The following subsections examine performance trade-offs across individual dimensions.

Table 20 provides a comprehensive cross-domain comparison consolidating accuracy (RMSE and $R^{2}$), computational efficiency (training time), temporal generalization ($\Delta R^{2}$ gap), interpretability (feature stability ρ), and robustness (drift degradation) for all six algorithms across three domains. This unified view reveals that no single algorithm dominates across all metrics: modern GBDT methods (XGBoost, LightGBM, and CatBoost) occupy the accuracy–speed Pareto front, while classical boosting (AdaBoost and GBM) provides superior temporal robustness and domain-specific drift resistance. The following subsections detail these trade-offs across individual evaluation dimensions, culminating in actionable deployment guidelines.

#### 7.6.1. Accuracy–Speed Trade-Offs Across Domains

**Figure 7:**
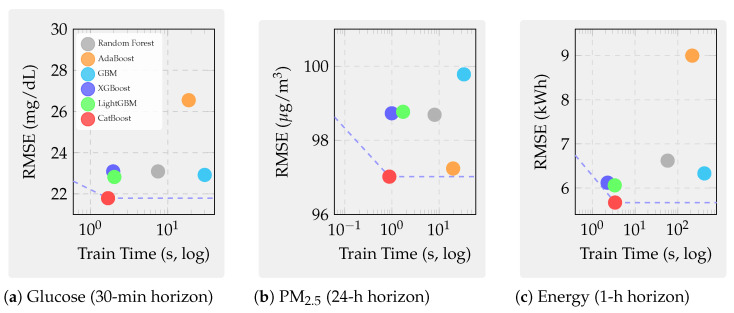
Cross-domain performance-efficiency Pareto fronts with dashed envelopes.

Figure 7 reveals consistent Pareto dominance by modern GBDT across all three forecasting domains (Table 20 provides numerical details). CatBoost achieves optimal accuracy in glucose (21.79 mg/dL and $R^{2}$ = 0.838; 1.69 s), PM_2.5_ (97.02 μg/m^3^ and $R^{2}$ = 0.078; 0.88 s), and energy (5.67 kWh and $R^{2}$ = 0.764; 3.41 s). Accuracy gains scale with domain predictability: high-$R^{2}$ regimes show 5–14% RMSE improvements over Random Forest (glucose and energy), while low-$R^{2}$ domains exhibit negligible algorithmic differentiation (PM_2.5_ spans only 2.8%). Classical methods (GBM and AdaBoost) universally train 10–100× slower without compensating accuracy benefits, confirming modern frameworks’ strict Pareto superiority for accuracy–speed trade-offs.

#### 7.6.2. Temporal Generalization: The Look-Ahead Bias Problem

**Figure 8:**
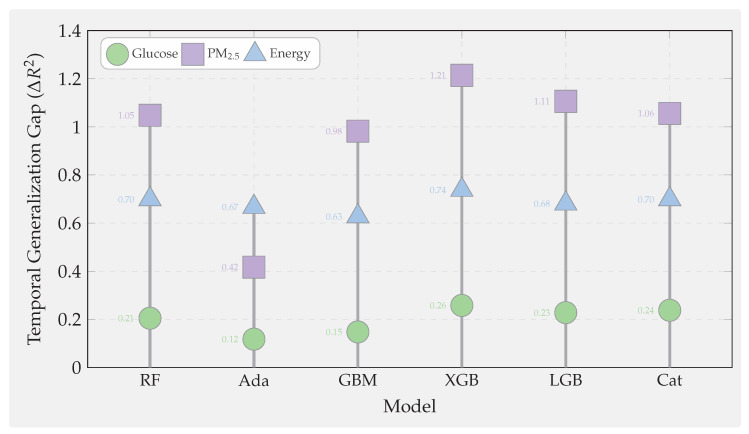
Cross-domain temporal generalization gaps. Each stem height represents $\Delta R^{2}=R_{\mathrm{random}}^{2}-R_{\mathrm{time}\text{-}\mathrm{series}}^{2}$.

Random CV systematically overestimates model performance across all three domains. However, the bias magnitude varies by forecasting context. Figure 8 visualizes temporal gaps ($\Delta R^{2}=R_{\mathrm{random}}^{2}-R_{\mathrm{time}\text{-}\mathrm{series}}^{2}$) as lollipop plots, where stem height represents gap magnitude and marker shapes distinguish domains: circles (glucose), squares (PM_2.5_), and triangles (energy).

PM_2.5_ exhibits dramatically larger temporal leakage. The purple squares cluster at heights 0.42–1.21, with AdaBoost maintaining the smallest gap (0.42) and XGBoost the largest (1.21). Modern GBDT methods (CatBoost 1.06, LightGBM 1.11, and XGBoost 1.21) demonstrate severe vulnerability to seasonal leakage: random CV allows models to memorize winter-high and summer-low pollution patterns that vanish when forecasting genuinely unseen future periods. These gaps indicate that random CV grossly overestimates forecasting skill. Models appearing to achieve $R^{2}$ =0.33–0.89 in development deliver negative $R^{2}$ in actual time-series deployment, revealing no genuine predictive ability.

Energy forecasting shows intermediate gaps. The blue triangles range from 0.63 to 0.74, reflecting hour-to-hour autocorrelation bias, although with smaller magnitude than PM_2.5_, due to weaker seasonal amplitude. GBM achieves the smallest gap (0.63), while XGBoost shows the largest (0.74). The visual contrast between tall purple squares (PM_2.5_) and shorter blue triangles (energy) illustrates domain-specific leakage severity. These 0.6–0.7 point gaps translate to models appearing to reach $R^{2}$ = 0.90–0.99 under random CV yet delivering only $R^{2}$ = 0.24–0.34 when forecasting future load profiles, a critical discrepancy for operational building management systems.

Glucose prediction demonstrates the best temporal robustness. The green circles cluster lowest (0.12–0.26) due to stronger physiological persistence and minimal seasonal structure. AdaBoost achieves the smallest gap (0.12), with modern GBDT showing progressively larger leakage: LightGBM (0.23), CatBoost (0.24), and XGBoost (0.26). Despite being smallest in absolute magnitude, these gaps carry clinical significance: models appearing to achieve $R^{2}$ = 0.94–0.96 in development deliver only $R^{2}$ = 0.68–0.74 in forward forecasting, sufficient to increase false-alarm rates in automated insulin delivery systems.

Cross-domain pattern: AdaBoost consistently minimizes temporal leakage. The lollipop visualization reveals a domain-invariant finding: AdaBoost maintains the shortest stems across all three contexts (glucose 0.12, PM_2.5_ 0.42, and energy 0.67), while XGBoost exhibits the tallest stems in glucose (0.26) and PM_2.5_ (1.21). This visual pattern confirms a fundamental trade-off: modern GBDT achieves superior baseline accuracy but sacrifices temporal robustness, whereas classical boosting prioritizes generalization stability over maximum performance. The consistent height ordering across marker shapes suggests practitioners must choose between CatBoost/LightGBM (accuracy-optimized) and AdaBoost/GBM (robustness-optimized), with optimal selection depending on whether deployment emphasizes absolute performance or temporal reliability.

These domain-specific gaps mandate universal adoption of time-series CV for sensor forecasting. Random CV systematically overestimates deployment performance: glucose models appearing to reach $R^{2}=0.94$ deliver only $R^{2}=0.70$ in practice (raising false-alarm rates in insulin delivery); PM_2.5_ models showing $R^{2}=0.87$ collapse to negative skill; energy models reporting $R^{2}=0.98$ achieve only $R^{2}=0.30$ when forecasting future loads. The consistent pattern across domains confirms that time-aware validation is not optional but mandatory for reliable sensor analytics deployment.

#### 7.6.3. Robustness Under Sensor Degradation

Robustness analysis reveals context-dependent failure modes under synthetic sensor perturbations. Table 21 summarizes maximum-intensity degradation (RMSE increase percentage) across three perturbation types and three domains.

Under Gaussian noise (random high-frequency fluctuations), AdaBoost and CatBoost consistently achieve the lowest degradation across all domains: glucose (4.9–5.2%), PM_2.5_ (0.0–0.6%), and energy (5.7–14.5%). Modern GBDT methods (XGBoost, LightGBM, and GBM) show moderate to high sensitivity in glucose (9.4–10.3%) and energy (22.6–27.0%) but remain robust in PM_2.5_ (0.1–1.2%) due to already dominant measurement noise. Random Forest maintains intermediate degradation (1.2–25.6% across domains), demonstrating that bagging provides reasonable but not optimal protection against stochastic perturbations.

Under impulse noise (intermittent dropouts and spikes), CatBoost and AdaBoost again dominate: glucose (69.6–89.7%), PM_2.5_ (1.6–3.1%), and energy (43.9–93.2%). Modern GBDT methods show substantially higher vulnerability: glucose (136.0–148.2%), PM_2.5_ (5.7–15.2% for LightGBM/XGBoost, although GBM reaches 15.2%), and energy (148.7–157.4%). Random Forest exhibits the worst degradation in glucose (149.2%) and energy (157.4%), confirming that bootstrap aggregation without explicit outlier rejection fails under corruption. The 2× robustness advantage CatBoost maintains over XGBoost/LightGBM (e.g., glucose 69.6% vs. 136.0–139.4%) stems from ordered boosting’s implicit outlier downweighting.

Under calibration drift (cumulative systematic bias), performance rankings reverse dramatically. GBM and XGBoost achieve the best stability in glucose (448.2–450.2%) and moderate stability in energy (537.1–551.4%), while CatBoost, LightGBM, and Random Forest show substantially higher degradation (glucose 558.7–579.7% and energy 488.4–585.8%). PM_2.5_ exhibits a different pattern: CatBoost maintains exceptional stability (12.0%), followed by LightGBM (25.2%) and XGBoost (57.5%), while classical methods fail catastrophically (AdaBoost 96.0%, Random Forest 103.7%, and GBM 166.3%). These domain-dependent differences suggest that drift robustness depends on interactions between tree depth, regularization strength, and baseline noise levels rather than any single algorithmic property.

The robustness synthesis reveals no universally superior algorithm. CatBoost excels under Gaussian and impulse noise but suffers under drift in physiological domains. GBM/XGBoost handle drift better but degrade severely under impulse corruption. AdaBoost maintains consistent moderate performance across perturbation types. Practitioners should match robustness profiles to their deployment conditions. For wireless CGM devices with dominant impulse noise, use CatBoost. For long-wear sensors without recalibration where drift dominates, use GBM/XGBoost. For well-maintained infrastructure with mainly Gaussian noise, select based on accuracy alone.

#### 7.6.4. Feature-Importance Stability and Interpretability

**Figure 9:**
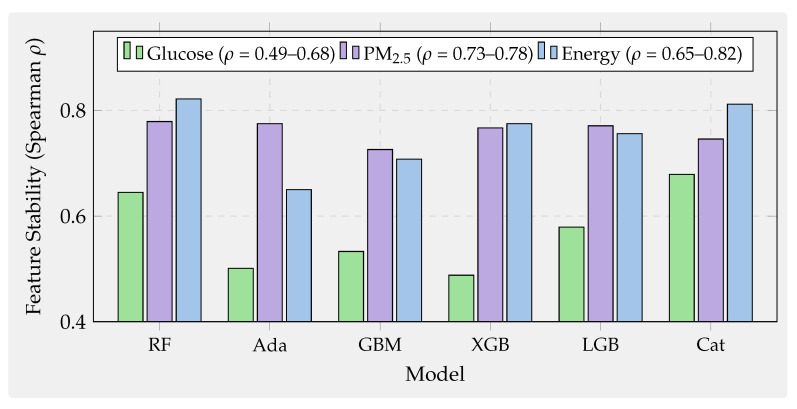
Cross-domain feature-importance stability (Spearman ρ of SHAP rankings).

Feature-importance stability (Spearman correlation of SHAP rankings across CV folds) exhibits domain-dependent patterns reflecting underlying signal structure. Figure 9 compares stability (ρ) across domains and algorithms.

PM_2.5_ forecasting exhibits the highest feature stability (ρ = 0.726–0.779) despite having the lowest predictive $R^{2}$ (less than 0.10). Random Forest achieves the best consistency (0.779), followed closely by AdaBoost (0.775) and LightGBM (0.771). This apparent paradox (high stability with low accuracy) arises because all models consistently identify the same weak but persistent meteorological features: recent PM_2.5_ lags, temperature, wind speed, and hour of day remain top-ranked across folds. Even though these features explain only a small fraction of total variance, their physical relevance produces stable SHAP rankings, valuable for policy applications, where attribution matters more than absolute forecasting skill.

Energy forecasting shows comparably high stability (ρ = 0.650–0.822), with Random Forest achieving the best consistency (0.822) and CatBoost close behind (0.812). Modern GBDT methods (XGBoost 0.775 and LightGBM 0.756) maintain reasonable stability, while AdaBoost exhibits the lowest (0.650) due to aggressive sample reweighting that shifts feature emphasis across folds. The high stability reflects deterministic drivers (outdoor temperature, occupancy schedules, and previous-hour load) that remain consistently influential regardless of training fold composition.

Glucose prediction displays the lowest feature stability (ρ = 0.488–0.679). This reflects complex physiological interactions. Feature importance depends on patient-specific factors like insulin sensitivity, meal timing, and activity patterns. CatBoost achieves the best stability (0.679), followed by Random Forest (0.645) and LightGBM (0.579). XGBoost exhibits the lowest consistency (0.488), suggesting that its aggressive gradient updates and deeper trees amplify fold-specific feature interactions. Despite moderate absolute correlations (0.5–0.7), qualitative inspection reveals that top-ranked features (CGM lags 1–3, insulin-on-board, hour of day, and recent carbohydrates) remain consistent, indicating acceptable interpretability for clinical deployment.

The stability analysis reveals an inverse relationship between predictive $R^{2}$ and feature stability: low-$R^{2}$ domains (PM_2.5_) show high stability from consistently weak signals, while high-$R^{2}$ domains (glucose and energy) show moderate stability due to richer feature interactions. Random Forest consistently achieves top-tier stability across all domains through bagging-based variance reduction, while modern GBDT methods (particularly XGBoost) sacrifice some interpretability consistency for marginal accuracy gains. For applications requiring transparent explanations (clinical decision support and policy attribution), Random Forest or CatBoost provide optimal stability–accuracy balance.

#### 7.6.5. Unified Deployment Guidelines

Building on the domain selection rationale (Section 7.1) and the empirical findings across glucose, PM_2.5_, and energy forecasting, we provide actionable recommendations indexed by problem characteristics rather than application labels. Practitioners should first characterize their sensor forecasting task along three dimensions: (1) intrinsic predictability ($R^{2}$ achievable with any method), (2) temporal structure (autocorrelation strength and seasonal amplitude), and (3) dominant noise mode (Gaussian, impulse, and drift). The following guidelines map these characteristics to optimal algorithm choices.

Table 20 summarizes the key trade-offs observed across all three forecasting domains. Based on this comprehensive cross-domain analysis, we provide the following actionable recommendations for sensor-based forecasting:

Model Selection by Domain Predictability:

- High-$R^{2}$ regimes (glucose $R^{2}>0.80$; energy $R^{2}>0.70$): Modern GBDT (CatBoost, LightGBM, and XGBoost) consistently deliver 5–15% RMSE improvements over Random Forest with training times under 4 s. CatBoost provides optimal accuracy–stability balance; XGBoost maximizes speed (approximately 2 s); LightGBM offers intermediate performance.
- Low-$R^{2}$ regimes (PM_2.5_ $R^{2}<0.10$): Model choice matters less than feature engineering and exogenous inputs. CatBoost and AdaBoost achieve marginally lower error (approximately 0.6–2.8% relative improvement), but absolute gains remain modest. Prioritize computational efficiency (XGBoost 0.99 s; CatBoost 0.88 s) and invest in numerical weather prediction integration rather than algorithm optimization.

Validation Strategy:

- Time-series CV is mandatory for all sensor forecasting applications. Random CV systematically overestimates performance by $\Delta R^{2}$ = 0.12–0.26 (glucose), 0.42–1.21 (PM_2.5_), and 0.63–0.74 (energy), corresponding to deployment failures ranging from increased false alarms (glucose) to negative skill (PM_2.5_).
- For applications requiring maximum temporal robustness (e.g., long-term model deployment without retraining), prioritize AdaBoost or GBM despite their lower baseline accuracy and higher computational cost. These methods achieve 50–70% smaller temporal gaps than modern GBDT in PM_2.5_ and energy forecasting.

Robustness Priorities by Deployment Context:

- Wireless/mobile sensors (impulse noise dominant): Deploy CatBoost or AdaBoost (2× lower degradation than modern GBDT at 40% corruption).
- Long-wear sensors without recalibration (drift dominant): Deploy GBM or XGBoost for glucose/energy (approximately 450% vs. 550–580% degradation); deploy CatBoost for PM_2.5_ (12% vs. 57–166% degradation).
- Well-maintained infrastructure (Gaussian noise dominant): Select based on accuracy and speed; robustness differences remain modest (less than 10% degradation span).

Interpretability Requirements:

- Clinical/policy applications requiring stable explanations: Deploy Random Forest (highest stability across energy/PM_2.5_, ρ = 0.78–0.82) or CatBoost (best stability in glucose, ρ=0.68).
- Performance-critical applications: Deploy CatBoost or LightGBM (optimal accuracy with acceptable stability, ρ = 0.65–0.81).
- Avoid XGBoost for interpretability-critical applications (lowest stability in glucose ρ = 0.49; moderate in other domains).

Computational Constraints:

- Edge devices requiring sub-second updates: Deploy XGBoost (fastest training: 1.0–2.3 s) or CatBoost (best accuracy at approximately 1.7–3.4 s).
- Cloud/server deployment with ample compute: CatBoost or LightGBM provide optimal accuracy; training time becomes negligible.
- Avoid classical methods (GBM 30–417 s and AdaBoost 19–217 s) unless specific robustness requirements (temporal gaps and drift stability) mandate their use.

In summary, no single algorithm dominates across all evaluation axes. Modern GBDT methods (XGBoost, LightGBM, and CatBoost) provide the best default choice for accuracy–speed trade-offs, but practitioners must carefully consider temporal generalization gaps, robustness profiles, and interpretability requirements when selecting models for production deployment. The optimal choice depends on domain predictability, expected sensor degradation modes, validation strategy, and operational constraints rather than benchmark accuracy alone.

#### 7.6.6. Generalization Mechanisms and Cross-Task Transferability Analysis

The preceding cross-domain analysis revealed both consistent patterns (modern GBDT dominates accuracy–speed trade-offs universally) and context-dependent variations (robustness rankings reverse across noise modes). Understanding why certain algorithmic properties generalize across tasks while others remain domain-specific enables principled extrapolation to new sensor analytics applications beyond the three benchmarked domains.

Training speed advantages exhibited remarkable cross-task consistency. Modern GBDT frameworks achieved 10–100× speedup over classical methods (GBM and AdaBoost) across all three domains, with relative rankings stable: XGBoost fastest (0.99–2.28 s), followed by CatBoost (0.88–3.41 s) and LightGBM (1.72–3.35 s), versus classical methods (GBM 29.93–416.49 s; AdaBoost 18.62–216.87 s). This consistency stems from architectural properties independent of data characteristics:

- Histogram-based split finding (LightGBM, XGBoost, and CatBoost) reduces computational complexity from O(nd) to $O(n_{\mathrm{bins}}d)$ regardless of domain.
- Column-block parallelization exploits multi-core hardware uniformly across tabular sensor data.
- GPU acceleration scales predictably with dataset size, not domain semantics.

Computational advantages discovered in glucose, PM_2.5_, and energy forecasting transfer directly to any tabular sensor regression task. Practitioners can confidently deploy modern GBDT and expect similar speed gains in new domains (e.g., industrial vibration monitoring, smart agriculture, and traffic prediction).

In contrast, accuracy advantages showed systematic dependence on intrinsic predictability ($R^{2}$ ceiling achievable by any method):

- High-$R^{2}$ regime (glucose $R^{2}=0.82$; energy $R^{2}=0.76$): Modern GBDT achieved 5–15% RMSE improvement over Random Forest and classical boosting through superior regularization and categorical handling.
- Low-$R^{2}$ regime (PM_2.5_ $R^{2}=0.05$): Algorithmic differences compressed to <3% RMSE span; all methods saturated at meteorological uncertainty limits.

This pattern reflects a fundamental principle: when signal-to-noise ratio collapses, algorithmic sophistication yields diminishing returns. The mechanism generalizes via

$$\Delta_{\mathrm{RMSE}}\propto {\left(R_{\mathrm{ceiling}}^{2}\right)}^{\alpha },\;\alpha \approx 0.5--1.0,$$

where $\Delta_{\mathrm{RMSE}}$ is the improvement from advanced methods over baselines.

For new sensor domains, practitioners can estimate transferable accuracy gains by first establishing $R^{2}$ ceiling via simple baselines (linear regression and Random Forest). If $R^{2}>0.70$, expect modern GBDT to deliver substantial improvements (5–15%); if $R^{2}<0.20$, expect marginal differentiation (<5%), suggesting investment in feature engineering over algorithm tuning.

Robustness rankings exhibited domain-specific reversals tied to dominant noise characteristics rather than application semantics:

- Impulse noise: CatBoost and AdaBoost consistently outperformed across glucose (69.6% vs. 136–149% degradation) and energy (43.9% vs. 148–157%), but PM_2.5_ differences compressed (1.6% vs. 5.7–15.2%) due to already dominant measurement variance.
- Calibration drift: GBM/XGBoost excelled in glucose (448–450%), but CatBoost dominated PM_2.5_ (12% vs. 58–166%), reflecting interactions between tree depth, baseline noise, and drift slope.

The generalization mechanism operates through algorithmic-noise interaction rather than domain identity. CatBoost’s ordered boosting effectively downweights transient outliers (impulse noise) but struggles with systematic bias accumulation (drift) in low-noise environments.

New applications should characterize expected noise profiles (impulse-dominated wireless sensors, drift-prone long-wear devices, and Gaussian-dominated calibrated instruments) rather than domain labels. Match algorithmic strengths to noise mode: CatBoost/AdaBoost for impulse-prone systems; GBM/XGBoost for drift-vulnerable long-term deployments.

Temporal generalization gaps exhibited consistent algorithmic ordering but domain-dependent magnitudes:

- AdaBoost consistently minimized gaps across glucose ($\Delta R^{2}=0.118$), PM_2.5_ (0.417), and energy (0.667).
- Modern GBDT showed larger gaps in all domains (XGBoost: 0.258, 1.214, and 0.738), but absolute magnitude scaled with autocorrelation strength and seasonal amplitude.

The mechanism: aggressive gradient updates and deeper trees (modern GBDT) exploit short-term correlations more readily, causing steeper performance degradation when random CV breaks temporal dependencies.

Transferability heuristic: For new time-series forecasting tasks, expect AdaBoost/GBM to provide 20–50% smaller temporal gaps than modern GBDT. If infrequent retraining is required (monthly updates and concept drift monitoring), prioritize classical methods despite baseline accuracy disadvantages.

Stability rankings (Random Forest > CatBoost > LightGBM > XGBoost) replicated across domains, driven by algorithmic properties:

- Bagging variance reduction (Random Forest) stabilizes importance uniformly.
- Ordered boosting (CatBoost) reduces fold-specific overfitting consistently.
- Aggressive gradient descent (XGBoost) amplifies feature-interaction sensitivity.

Absolute stability magnitudes varied by signal strength (PM_2.5_ highest ρ = 0.73–0.78; glucose lowest ρ = 0.49–0.68), but relative rankings remained invariant.

Transferability: For interpretability-critical applications (clinical and regulatory), Random Forest and CatBoost provide consistently stable explanations regardless of domain. XGBoost sacrifices stability for marginal accuracy gains—acceptable for performance-critical explanation-optional contexts.

To predict algorithm suitability in unseen sensor analytics tasks, practitioners should characterize the following:

1. Intrinsic predictability: Run baseline models to estimate $R^{2}$ ceiling → determines accuracy gain magnitude.
2. Temporal structure: Quantify autocorrelation decay and seasonal periods → determines temporal gap severity.
3. Dominant noise mode: Identify primary degradation mechanism (impulse, drift, or Gaussian) → determines robustness priorities.
4. Interpretability requirements: Assess stability needs for explanations → determines algorithm-stability mapping.

This four-dimensional characterization enables evidence-based algorithm selection extrapolated from the glucose–PM_2.5_–energy benchmark to arbitrary sensor forecasting contexts, avoiding trial-and-error exploration across the algorithmic design space.

## 8. Practical Decision Framework

Building on the cross-domain synthesis (Section 7.6), which established unified recommendations for algorithm selection based on domain predictability, robustness requirements, and temporal generalization, this section provides operational guidance for implementing and deploying boosting methods in production sensor analytics systems. We focus on hyperparameter configuration, evaluation protocols, deployment lifecycle management, and computational considerations that practitioners encounter when translating benchmark insights into working systems.

### 8.1. Hyperparameter Configuration Guidelines

Effective hyperparameter tuning requires balancing model capacity with generalization while respecting computational constraints. Based on our empirical results across glucose, PM_2.5_, and energy forecasting, we recommend the following configurations as robust starting points.

Learning rate and number of estimators: Conservative learning rates (0.01–0.05) paired with larger ensemble sizes (200–500 trees) provide stable convergence across diverse sensor domains [8]. Lower learning rates reduce sensitivity to individual tree errors and improve generalization to unseen temporal patterns, critical for time-series forecasting where validation folds may not fully represent deployment distributions. Use early stopping with patience of 20–50 rounds to prevent overfitting while maintaining training efficiency [8].

Implementation varies by framework: XGBoost typically requires 300–500 trees at learning rate 0.03 to reach convergence [12]; LightGBM’s leaf-wise growth strategy often achieves comparable error with 30–50% fewer trees (150–300 estimators) at the same learning rate, reducing both training time and memory footprint [22]. CatBoost benefits from slightly higher learning rates (0.05–0.08) due to ordered boosting’s built-in regularization, typically converging with 200–400 trees [45]. For low-predictability regimes (PM_2.5_; $R^{2}<0.10$), limit ensembles to 100–200 trees as additional capacity provides negligible accuracy gains while increasing overfitting risk.

Monitor validation curves during training: If training error continues decreasing while validation error plateaus or increases, reduce learning rate by 50% and increase tree count proportionally. For edge deployment with strict latency constraints (inference under 10 ms), reduce ensemble size to 50–100 trees and increase learning rate to 0.08–0.10, accepting 2–5% accuracy degradation for 3–5× faster prediction [56].

Tree depth and structural regularization: Maximum tree depth controls model capacity and interaction complexity. Shallow trees (depth 3–5) suffice for most sensor forecasting tasks, where predictive features exhibit primarily additive or pairwise interactions: glucose lags plus insulin-on-board, temperature plus hour of day for energy loads, and wind speed plus PM_2.5_ lags for air quality. Depths exceeding 8 risk overfitting to fold-specific noise unless training sets contain over 100,000 samples with rich feature interactions.

Domain-specific tuning: Glucose prediction with 113 features benefits from depth 4–5 to capture insulin–carbohydrate–activity interactions; PM_2.5_ with sparse meteorological features converges at depth 3–4; heterogeneous building portfolios with diverse HVAC systems may require depth 6-8 to model equipment-specific load profiles. Start with depth 4 and increase by 1 if validation $R^{2}$ improves by over 2% on held-out folds.

Apply $\ell_{2}$ regularization (lambda 0.5–2.0 for XGBoost/LightGBM; 1.0–5.0 for CatBoost) to penalize complex trees [12]. For domains with known causal relationships, enforce monotonic constraints: glucose should increase monotonically with carbohydrate intake; building load should increase with cooling degree days; PM_2.5_ should decrease with wind speed [12,156]. These constraints improve interpretability, prevent spurious inversions in extrapolation regimes, and often yield 1–3% validation accuracy gains by encoding physical priors.

Minimum child weight (min_child_weight in XGBoost; min_child_samples in LightGBM) prevents splits on noise: set to 5–10 for small (under 10,000 samples), 20–50 for medium (10,000–100,000), and 50–100 for large datasets. PM_2.5_ forecasting benefits from higher values (30–50) due to high measurement noise; glucose requires lower values (5–10) to capture rare hypoglycemic/hyperglycemic patterns.

Subsampling strategies: Row and feature subsampling reduce overfitting and training time. Set row subsampling to 0.6–0.8 for most applications [12,22]. Lower values (0.5–0.6) benefit high-noise domains (PM_2.5_) by reducing sensitivity to outlier samples; higher values (0.7–0.9) suit cleaner physiological signals (glucose and energy) where each sample provides valuable information.

Feature subsampling (colsample_bytree in XGBoost; feature_fraction in LightGBM) mitigates multicollinearity [12,22]. For meteorological features with high redundancy (temperature, dew point, and humidity often correlate at 0.8–0.95), use aggressive subsampling (0.3–0.5) to force diverse tree structures. For engineered feature sets with low correlation (glucose: separate insulin, carbohydrate, activity, and circadian features), use conservative subsampling (0.6–0.8) to allow each tree access to multiple information sources. Energy forecasting with calendar features benefits from per-level subsampling (colsample_bylevel 0.5–0.7) to ensure occupancy and weather features contribute at different tree depths.

Categorical feature handling: CatBoost’s ordered target statistics provide the most robust categorical encoding, eliminating the need for manual preprocessing [45]. For station IDs (PM_2.5_), building types (energy), or patient identifiers (glucose), simply mark features as categorical and allow CatBoost to learn optimal split strategies. This approach automatically handles rare categories, prevents target leakage, and adapts to class imbalances without requiring separate validation-aware encoding pipelines.

For XGBoost and LightGBM, implement leakage-safe target encoding within CV folds: compute category means on training folds only, apply smoothing (blend with global mean using factor 10–50), and add Gaussian noise (standard deviation 0.01–0.05 times target range) to prevent memorization [45]. Never use test-fold information during encoding. Alternatively, use label encoding for ordinal categories (hour of day: 0–23; month: 1–12) or one-hot encoding for low-cardinality features (building type: 3–5 classes), accepting the dimensionality increase.

For high-cardinality categoricals (hundreds of building IDs; thousands of sensor locations), prefer CatBoost or use hash encoding (feature hashing) with XGBoost/LightGBM to limit feature explosion. Hash 10,000 unique IDs into 100–200 bins, accepting minor collision-induced noise as a regularization benefit. Monitor for cold-start problems: new categories unseen during training should fall back to global means rather than causing prediction failures.

### 8.2. Evaluation Protocol and Metrics

Rigorous evaluation requires time-aware validation, appropriate metrics, and computational profiling to ensure reported performance generalizes to deployment conditions.

As established in Section 7.6, random CV systematically overestimates sensor forecasting performance through temporal leakage [67,68]. Implement forward-chaining time-series splits: train on months 1–6, validate on month 7; train on months 1–9, validate on month 10; continue until data exhaustion. Maintain minimum validation fold sizes of 10% of total samples to ensure statistical reliability.

Use 5-fold time-series CV for moderate datasets (10,000–50,000 samples), expanding to 10-fold for large datasets (over 100,000 samples) where computational cost permits [68]. For multi-site deployments (12 Beijing stations; 10 buildings), perform nested CV: outer loop iterates over sites for spatial generalization assessment; inner loop performs time-series splitting within each site for hyperparameter tuning. Report both within-site (interpolation) and across-site (extrapolation) errors to characterize model transferability.

Respect domain-specific temporal structure: glucose forecasting should validate on complete days to capture circadian effects; energy forecasting requires full weeks to assess weekday–weekend transitions; PM_2.5_ needs seasonal validation windows (winter vs. summer) to test performance across meteorological regimes. Never split within autocorrelation timescales: avoid training on hour 23 and validating on hour 24 of the same day.

Report a comprehensive metric suite covering accuracy, calibration, and temporal generalization. Accuracy metrics: RMSE (penalizes large errors, appropriate for safety-critical glucose forecasting), MAE (robust to outliers, suitable for noisy PM_2.5_), and $R^{2}$ (interpretable variance explanation). For domains with heterogeneous scales (multi-building portfolios), add normalized metrics: CVRMSE (coefficient of variation of RMSE) or MAPE (mean absolute percentage error), ensuring fair comparison across sites [26].

Always report $\Delta R^{2}=R_{\mathrm{random}}^{2}-R_{\mathrm{ts}}^{2}$ to quantify look-ahead bias magnitude [67,68]. Values exceeding 0.2 indicate substantial overfitting to temporal structure; values exceeding 0.5 (common in PM_2.5_ and energy) signal that random CV should never be trusted for model selection. Use this gap as a red flag during peer review: publications reporting only random CV results without temporal validation warrant skepticism.

For glucose, report time-in-range (percentage of predictions within 70–180 mg/dL), hypoglycemia detection rate (sensitivity for glucose under 70 mg/dL), and clinical utility metrics (Clarke Error Grid zones A + B percentage) [9]. Standard RMSE thresholds: under 20 mg/dL excellent, 20–25 mg/dL acceptable, and over 30 mg/dL unsuitable for insulin dosing. For PM_2.5_, include exceedance detection (precision/recall for daily average over 75 μg/m^3^ WHO guideline), early warning lead time (hours before pollution episode), and false alarm rate. For energy, report peak demand error (RMSE during top 10% load hours, critical for grid planning), load factor preservation, and demand-response accuracy [26].

Report training time (seconds on specified CPU/GPU), inference latency (milliseconds per sample), memory footprint (MB for model serialization), and model size (MB for deployment package) [56]. Compare against baseline: “CatBoost trains 15× faster than GBM with 2% better accuracy” provides actionable information; “CatBoost achieves RMSE 21.79” in isolation does not.

Test significance of performance differences using paired *t*-tests or Wilcoxon signed-rank tests across CV folds, reporting *p*-values for key comparisons. Differences under 2% RMSE rarely achieve significance (*p* under 0.05) with typical fold counts (5–10), indicating that algorithm rankings may reflect random variation rather than true superiority. When reporting “best model,” include confidence intervals (CIs) (e.g., “CatBoost RMSE 21.79 ± 3.01 mg/dL, 95% CI [20.4, 23.2], significantly better than AdaBoost p=0.03”).

Ensure reproducibility: fix random seeds for data splitting, tree construction, and subsampling; specify software versions (XGBoost 1.7.x, LightGBM 3.3.x, and CatBoost 1.2.x); document preprocessing steps (scaling, imputation, and feature engineering) with executable code. Provide trained models and validation predictions to enable independent verification. For production systems, version control hyperparameter configurations and track model lineage from training data through deployment.

### 8.3. Deployment Considerations

Effective deployment extends beyond initial model training to encompass updating strategies, performance monitoring, and interpretability integration.

Sensor streams exhibit temporal drift from equipment aging, seasonal shifts, behavior changes, and policy interventions, requiring scheduled model updates [6]. Our robustness analysis (Section 7.6) demonstrates that static models suffer catastrophic degradation under calibration drift (450–580% RMSE increase across domains), mandating periodic retraining even for well-maintained sensors.

Establish domain-specific retraining schedules: For glucose (personalized healthcare), weekly to biweekly retraining captures changing insulin sensitivity, activity patterns, and meal preferences. Trigger immediate retraining when 7-day rolling RMSE increases over 20% above baseline, indicating regime shifts (illness, medication changes, and sensor replacement). For PM_2.5_ (air quality), seasonal retraining (quarterly) accommodates heating-season vs. summer meteorology and evolving emission inventories. Monitor forecast skill degradation during transitional months (March–April; September–October); retrain when skill drops below 0.5× persistence baseline [157]. For energy (buildings), conduct monthly retraining during occupancy transitions (academic semesters and seasonal schedules), weekly during stable periods. Retrain immediately after HVAC retrofits, setpoint changes, or occupancy model updates that invalidate learned load patterns.

For continuous high-frequency streams, consider incremental learning: online gradient boosting methods [35,146] update existing trees with new batches rather than retraining from scratch, reducing computational costs by 10–50× while maintaining accuracy within 2–5% of full retraining. However, incremental methods accumulate bias over time; perform full retraining every 3–6 months to reset model state [6].

Implement automated monitoring to detect distribution shifts. This prevents accuracy from degrading to unacceptable levels [6]. Track three complementary metrics: Error tracking (compute 7-day rolling RMSE and compare against training-fold baselines; alert when rolling error exceeds 1.5× baseline for 3 consecutive days), feature distribution monitoring (log mean, standard deviation, and quantiles for all input features; compare weekly distributions against training statistics using Kolmogorov–Smirnov tests; *p*-values below 0.01 flag potential covariate shift), and SHAP pattern stability (recompute feature importance rankings monthly using SHAP values on recent data; compare against training-fold rankings using Spearman correlation; ρ dropping below 0.6 indicates feature relationships have shifted) [6,29].

Trigger retraining automatically when any two of these three indicators cross thresholds simultaneously. Single-indicator alerts often reflect measurement noise or transient events; multiple concurrent alerts signal genuine model degradation requiring intervention.

Integrate SHAP analysis into operational workflows for transparency and debugging [29]. For global explanations, compute mean absolute SHAP values across all predictions to rank feature importance. For policy applications (PM_2.5_ attribution to wind, temperature, and emissions), report top-five features with quantified contributions (e.g., “wind speed explains 23% of model variance, hour-of-day 18%”). Use SHAP summary plots to visualize feature effect distributions, demonstrating whether relationships are linear (diagonal bands) or complex (scattered clouds).

For local explanations (case-level decisions), generate SHAP waterfall plots showing per-feature contributions to individual forecasts [29]. Clinicians can verify that hypoglycemia alerts stem from low recent glucose and high insulin-on-board rather than spurious correlations. Building managers can confirm that demand-response opportunities are real. They arise from genuine low-occupancy periods, not sensor errors.

Enforce monotonic constraints to encode known causal directions during training [12,156]: Glucose increases with carbohydrates (monotone_constraints: [1] for carbohydrate features); building load increases with cooling degree days (monotone_constraints: [1] for temperature features); PM_2.5_ decreases with wind speed (monotone_constraints: [−1] for wind features). These constraints prevent physically implausible inversions during extrapolation, improving stakeholder trust and reducing debugging burden when predictions appear anomalous.

For regulated domains (clinical decision support under FDA oversight and financial applications under model governance), maintain SHAP explanation archives: Store feature contributions for every prediction, enabling post hoc audits when model decisions are questioned. Accept 5–10% computational overhead (SHAP calculation adds 1–2 ms per inference) as necessary cost for transparent AI systems [156].

### 8.4. Implementation Recommendations

XGBoost offers a mature ecosystem with extensive documentation, integration with scikit-learn pipelines, and stable APIs [12]. It is the strongest choice for production systems requiring long-term maintainability and community support. LightGBM is optimized for speed and memory efficiency, ideal for large-scale multi-site deployments or frequent retraining scenarios [22]. Histogram-based algorithms reduce training time by 30–60% versus XGBoost without accuracy loss. CatBoost provides the simplest handling of categorical features, eliminating preprocessing complexity for mixed numeric-categorical sensor data [45]. Ordered boosting provides marginal accuracy improvements (1–3%) in heterogeneous domains. Select when categorical feature engineering overhead exceeds training time savings or when marginal accuracy gains justify additional computational cost.

Modern GBDT scales efficiently to 16–32 cores via shared-memory parallelism. For edge deployments (on-device retraining for personalized glucose models), target ARM-based systems (Raspberry Pi 4, NVIDIA Jetson) running LightGBM with 4–8 threads, achieving sub-10 s training for 10,000-sample datasets [56]. Quantize models to int8 precision (4× smaller; 2–3× faster inference) via post-training quantization, accepting under 1% accuracy degradation. XGBoost and LightGBM support CUDA/OpenCL acceleration, reducing training time from minutes to seconds for datasets exceeding 100,000 samples [12,22]. On NVIDIA A100 GPUs, expect 20–40× speedup for histogram construction and 10–15× for full training pipelines (including data transfer overhead). However, GPU benefits diminish for small datasets (under 50,000 samples) where CPU vectorization suffices.

Export trained models to ONNX Runtime for optimized inference (2–5× faster than native Python APIs), achieving under 1 ms latency for 100-tree ensembles on modern CPUs. Prune trees with near-zero SHAP contributions (bottom 20% by importance), recovering 15–25% memory and 10–20% inference speedup with under 0.5% accuracy loss [29].

Leverage AutoML frameworks to automate hyperparameter tuning and model selection while respecting time-series validation constraints. Optuna offers flexible Bayesian optimization supporting custom objectives, early stopping, and parallel search [131]. Run 100–300 trials targeting 2–5% RMSE improvement over default configurations, accepting 2–10× computational overhead versus manual tuning. AutoGluon provides end-to-end AutoML, supporting automatic feature engineering, multi-layer stacking, and ensemble selection [158]. Specify time-series splitter via fold_strategy parameter; enable GPU acceleration for large datasets; set time budget (1–6 h) based on deployment urgency. Typically achieves within 1–2% of expert-tuned performance with zero manual effort.

Integrate AutoML into CI/CD pipelines: automatically retune models monthly as new data arrives, selecting configurations that maintain accuracy while meeting latency SLAs [131,158]. Archive tuning histories to track hyperparameter drift over time, identifying when default configurations become suboptimal due to changing data distributions.

For healthcare (HIPAA) and consumer applications (GDPR), implement privacy-preserving ML workflows. Federated learning trains models on-device (patient smartphones and building controllers) without centralizing raw sensor data [57]. Aggregate only model gradients or tree structures across devices. XGBoost supports federated training via encrypted gradient exchange; expect 2–10× training overhead and 5–15% accuracy degradation versus centralized training. Suitable for glucose prediction (personalized models per patient) where data centralization poses privacy risks or regulatory barriers.

Differential privacy adds calibrated noise to gradients during training to bound information leakage about individual samples [57]. Use privacy budget (epsilon equals 1–10 for moderate protection), balancing privacy guarantees against utility loss. DP-SGD variants for boosting achieve epsilon equals 5 privacy with under 5% accuracy degradation on datasets exceeding 10,000 samples. Required for publishing aggregate models trained on protected health information.

Accept accuracy–privacy trade-offs transparently: Document degradation and justify based on regulatory requirements [57]. For non-sensitive applications (building energy and air quality), prioritize accuracy and deploy centralized models without privacy overhead.

## 9. Open Challenges and Future Directions

Despite significant progress in modern gradient boosting for sensor analytics, our cross-domain evaluation reveals fundamental limitations requiring continued research. This section identifies critical challenges and proposes directions for next-generation sensor monitoring systems.

### 9.1. Uncertainty Quantification and Calibration

Current boosting implementations produce point predictions lacking uncertainty estimates that are essential for risk-aware decision-making. Clinical applications like glucose monitoring require confidence intervals to distinguish genuine alerts from uncertain predictions near safety thresholds. Similarly, air-quality forecasting needs probabilistic outputs for graduated alert levels, and building-energy predictions require intervals for robust demand-response scheduling.

Our cross-domain analysis reveals a critical disconnect: models can exhibit stable feature importance rankings while delivering poor predictive performance, suggesting overconfidence in unreliable forecasts. Calibrated uncertainty quantification would flag these low-confidence regimes, preventing stakeholders from over-trusting model outputs.

Recent advances in quantile gradient boosting and conformal prediction offer promising approaches [63,159], but challenges remain in maintaining computational efficiency for real-time applications. Future research should develop uncertainty quantification methods for sensor time-series. These methods must respect temporal autocorrelation, handle nonstationary distributions, and scale to edge deployment constraints. Uncertainty-aware model selection could prevent deploying models with stable interpretability metrics but negative predictive skill.

### 9.2. Physics-Informed and Hybrid Learning

Pure data-driven boosting ignores valuable domain knowledge from decades of scientific research in physiology, atmospheric chemistry, and building thermodynamics. Our results demonstrate that statistical learning alone cannot infer complex physical processes from local sensor observations, particularly for atmospheric dispersion and glucose–insulin pharmacokinetics.

Hybrid architectures combining physics-based simulations with gradient boosting corrections for local patterns and sensor biases could bridge this gap [157]. Physics-informed neural networks (PINNs) encode differential equations as training loss regularization [160], but extending these principles to tree-based boosting requires novel formulations. While monotonic constraints provide limited physics encoding [12], they cannot represent complex differential constraints or conservation laws.

Promising directions include the following: (1) physics-guided feature engineering where domain models generate derived features, (2) hybrid loss functions penalizing violations of physical constraints, and (3) multi-fidelity learning combining statistical models with physics simulations via transfer learning [4]. Integrating physics-based priors into gradient boosting could substantially improve generalization. This is especially valuable for sparse data regimes and extrapolation beyond training distributions.

### 9.3. Handling Nonstationarity and Calibration Drift

Our robustness analysis reveals that calibration drift dominates other perturbation types, causing catastrophic degradation across all domains. Systematic sensor bias accumulation produces failure modes that current static tree ensembles cannot self-correct without explicit recalibration or adaptive retraining. This has profound implications for long-term deployments: continuous glucose monitors, air-quality networks, and building meters all accumulate drift that static models cannot accommodate.

Continual learning approaches that accumulate knowledge across time periods without catastrophic forgetting offer promising alternatives [161]. For sensor analytics, continual boosting must balance plasticity and stability. The challenges include determining when distribution shifts warrant model updates versus temporary anomalies and efficiently updating ensembles without full retraining. Online gradient boosting methods reduce computational costs but may accumulate bias over extended periods [35,146].

Meta-learning approaches could enable rapid personalization with limited data. These approaches learn how to adapt models across related sensor deployments [162]. Transfer learning from large pretrained ensembles to new sensors, adaptive learning rates responding to detected drift, and automated drift detection using principled statistical tests represent critical directions for robust long-term sensor analytics [6].

### 9.4. Privacy-Preserving Distributed Learning

Healthcare monitoring and building-energy management face strict privacy regulations preventing centralized data aggregation, while model quality improves with larger training sets spanning diverse patients, climates, and building types. Federated learning enables collaborative training across distributed sensors without raw data sharing [57], but adapting gradient boosting to federated settings faces unique challenges.

Tree structures are discrete and difficult to aggregate compared to neural network weight averaging. Secure aggregation protocols add communication overhead that is problematic for IoT sensors with limited bandwidth. Differential privacy mechanisms protecting individual privacy introduce noise that may degrade accuracy. Recent proposals include ensemble approaches, where each client contributes trees to a global ensemble [163], and knowledge distillation, where distributed models train a global student model [164].

Communication-efficient protocols, privacy–utility trade-offs, and handling heterogeneous client data distributions require continued research. Personalized federated learning combining shared global knowledge with local adaptation could balance privacy, performance, and fairness for next-generation distributed sensor analytics [57].

### 9.5. Interpretability Beyond Feature Importance

Our analysis reveals that consistent feature rankings do not guarantee predictive reliability or actionable insights. Models can reliably identify the same correlations across CV folds while failing to learn genuinely predictive relationships for forward forecasting. This paradox suggests that current interpretability methods measure feature usage consistency rather than causal or predictive validity.

For clinical and operational deployment, stakeholders require explanations beyond feature contributions. Clinicians need counterfactual reasoning quantifying intervention effects. Building managers need actionable recommendations for demand response. Current SHAP explanations describe model behavior without prescribing interventions or quantifying action consequences.

Causal inference methods identifying truly manipulable features, counterfactual explanation techniques, and natural language generation translating technical outputs into stakeholder-appropriate narratives represent critical directions [156]. For safety-critical applications, explanations must include confidence assessments distinguishing high-confidence predictions from uncertain estimates. Integrating uncertainty quantification with explainable AI could provide the transparent, actionable, and trustworthy decision support required for responsible sensor analytics deployment. Future explanation methods should be validated on held-out temporal folds rather than random samples to ensure robustness to distribution shift [29].

## 10. Conclusions

This cross-domain survey systematically evaluated boosting algorithms for sensor analytics across continuous glucose monitoring, urban air-quality forecasting, and building-energy prediction. Our analysis spans the physiological, environmental, and infrastructure sensing domains. We quantified fundamental trade-offs between accuracy, temporal robustness, and sensor-degradation resilience. These evaluations employed realistic deployment constraints to assess computational efficiency.

First, algorithmic sophistication yields diminishing returns when domain predictability collapses. Modern GBDT methods deliver substantial improvements in high-signal regimes like glucose and energy forecasting but converge to near-identical performance in low-predictability domains like PM_2.5_ forecasting, where stochastic meteorology dominates local sensor signals. Practitioners should estimate domain predictability upfront using persistence baselines before investing in algorithmic optimization; when signal-to-noise ratios collapse, prioritize exogenous data integration over boosting variants.

Second, temporal validation exposes severe look-ahead bias masked by random CV. Random shuffling substantially inflates apparent performance across all domains, with modern GBDT methods showing paradoxically higher vulnerability despite superior baseline accuracy. Models appearing highly accurate in development deliver substantially degraded performance in forward forecasting, and PM_2.5_ models memorize seasonal patterns that provide zero genuine predictive skill despite positive development metrics. Time-series validation is non-negotiable for sensor applications; publications reporting only random CV results systematically overestimate deployment performance and should face peer-review skepticism.

Third, calibration drift dominates sensor degradation with no universal solution. While Gaussian noise causes modest degradation and impulse corruption produces manageable effects, systematic bias accumulation catastrophically degrades all methods, with the effects remaining nearly constant across intensity levels. This confirms structural failure rather than magnitude-dependent vulnerability. Critically, no static ensemble self-corrects cumulative bias; practical deployments require scheduled retraining or continual learning approaches balancing plasticity with stability.

CatBoost provides the optimal default for high-accuracy applications requiring frequent retraining, achieving the best performance across all three domains with competitive training speed and superior impulse-noise robustness. LightGBM optimizes large-scale deployments through substantially faster convergence without accuracy loss. XGBoost maximizes inference speed but exhibits the largest temporal gaps, making it suitable only for high-frequency retraining scenarios. AdaBoost maintains the smallest temporal gaps despite its lower baseline accuracy, positioning it for remote sensors with infrequent recalibration where generalization stability outweighs absolute performance. Random Forest provides interpretable baselines for policy applications prioritizing transparent attributions over marginal accuracy gains.

Hyperparameter tuning requires domain awareness. Shallow trees suffice for sensor forecasting with predominantly additive interactions, while monotonic constraints improve accuracy and stakeholder trust. Aggressive feature subsampling benefits redundant meteorological data, whereas conservative values suit engineered feature sets. Robustness priorities dictate algorithm choice. Wireless sensors with impulse noise favor CatBoost or AdaBoost. Long-wear devices require drift-resistant variants, while well-maintained infrastructure can prioritize accuracy as degradation differences remain modest. Uncertainty quantification must address the confidence gap where models produce stable but unreliable predictions, revealed most clearly in PM_2.5_ forecasting. Physics-informed boosting should incorporate domain constraints to overcome residual variance limiting pure statistical learning. Continual learning frameworks must handle calibration drift’s structural failure through meta-learning and selective adaptation. Privacy-preserving federated approaches should accommodate spatial and temporal heterogeneity while maintaining differential privacy guarantees. Interpretability methods require advancement beyond feature rankings to counterfactual reasoning and causal validity assessment.

As sensor networks proliferate across smart cities, precision medicine, and climate monitoring, the trade-offs we quantified guide next-generation system design. No universal algorithm dominates all the evaluation axes; practitioners must navigate context-dependent choices balancing domain predictability, degradation modes, retraining constraints, and stakeholder requirements. This work provides both an empirical foundation for deploying boosting in sensor applications and a research agenda for advancing algorithmic capabilities. Future sensor analytics systems must integrate mandatory temporal validation, scheduled retraining for drift mitigation, uncertainty quantification for risk-aware decisions, and domain knowledge incorporation for physical consistency. Only through an honest appraisal of the current methods’ strengths and limitations can we build trustworthy sensor analytics addressing society’s pressing challenges in health, environment, and energy sustainability.

## Figures and Tables

**Table 1:** Comparison of modern GBDT frameworks for sensor analytics deployment.

Characteristic	XGBoost	LightGBM	CatBoost
Training Speed	Moderate	Fastest	Fast
Memory Usage	Moderate	Lowest	Low
Inference Latency	2–8 ms	1–6 ms	1–5 ms
Model Size	200–1000 KB	100–800 KB	50–500 KB
Categorical Handling	Manual	Manual	Native
Overfitting Resistance	Good	Moderate	Best
Edge Deployment	Moderate	Good	Excellent
Large-Scale Data	Good	Excellent	Good
GPU Support	Excellent	Excellent	Good
Interpretability	Good	Good	Excellent
Hyperparameter Sensitivity	Moderate	High	Low

**Table 2:** Interpretability and deployment trade-offs across boosting methods.

Characteristic	Classical	Modern GBDT	Recommendation
	(Ada, GBM)	(XGB, LGB, Cat)	
Direct Interpretability	High (shallow)	Low (deep)	Use SHAP for all
Feature Importance	Sparse	Distributed	Permutation for robustness
SHAP Computation	Fast	Moderate	TreeSHAP for all
Monotonic Constraints	Manual	Built-in	Leverage when available
Edge Deployment	Moderate	Excellent	Modern for IoT
Training Speed	Slow	Fast	Modern for production
Hyperparameter Sensitivity	Low	Moderate–High	AutoML for modern

**Table 3:** Computational overhead of adaptive boosting strategies relative to baseline GBDT.

Strategy	Training Cost	Inference Latency	Memory Footprint
Baseline GBDT	1×	1×	1×
Metaheuristic (PSO/GA)	10–50×	1×	1×
Online/Incremental	0.1–0.5× *	1–1.2×	0.5–1×
LSTM–Boosting	2–5×	1.5–2.5×	1.5–3×
CNN–Attention–Boosting	3–8×	2–4×	2–5×

* Per update; total cost depends on drift frequency and window size.

**Table 4:** Glucose prediction results (30-min horizon; 6 patients).

Model	RMSE (mg/dL)	MAE (mg/dL)	$R^{2}$	Train Time (s)
Random Forest	23.09 ± 2.86	16.15 ± 1.78	0.818 ± 0.061	7.47 ± 2.36
AdaBoost	26.55 ± 4.09	19.54 ± 3.37	0.762 ± 0.082	18.62 ± 8.74
GBM	22.92 ± 3.29	15.88 ± 2.06	0.821 ± 0.062	29.93 ± 12.22
XGBoost	23.09 ± 3.27	16.12 ± 2.01	0.819 ± 0.060	1.97 ± 0.78
LightGBM	22.82 ± 3.39	15.88 ± 2.08	0.823 ± 0.061	2.04 ± 0.47
CatBoost	**21.79 ± 3.01**	**15.03 ± 1.88**	**0.838 ± 0.056**	**1.69 ± 0.68**

Note: Bold values indicate best performance for each metric.

**Table 5:** Statistical significance tests for glucose prediction algorithms.

Friedman Omnibus Test						
Test Statistic	$\chi^{2}\left(5\right)=22.2$					
*p*-value	p<0.001					
Interpretation	Significant differences detected					
**Post hoc Nemenyi Pairwise Comparison ** ***p*** **-values**						
	Random Forest	AdaBoost	GBM	XGBoost	LightGBM	CatBoost
Random Forest	—	0.025 *	1.000	0.817	0.589	0.017 *
AdaBoost	0.025 *	—	0.025 *	0.045 *	0.006 **	<0.001 ***
GBM	1.000	0.025 *	—	0.817	0.589	0.017 *
XGBoost	0.817	0.045 *	0.817	—	0.440	0.009 **
LightGBM	0.589	0.006 **	0.589	0.440	—	0.064
CatBoost	0.017 *	<0.001 ***	0.017 *	0.009 **	0.064	—

Notes: Significance levels: *** p<0.001, ** p<0.01, and * p<0.05.

**Table 6:** RMSE degradation (%) under synthetic sensor faults across four intensity levels.

Model	Gaussian Noise				Impulse Noise				Calibration Drift			
	**Lv.1**	**Lv.2**	**Lv.3**	**Lv.4**	**Lv.1**	**Lv.2**	**Lv.3**	**Lv.4**	**Lv.1**	**Lv.2**	**Lv.3**	**Lv.4**
Random Forest	1.0	2.9	5.9	9.1	50.5	91.2	120.3	149.2	571.1	572.5	573.0	573.3
AdaBoost	**0.3**	**1.1**	**2.5**	**4.9**	27.7	50.6	69.9	89.7	477.0	478.3	478.8	479.0
GBM	1.4	3.1	6.5	10.3	53.1	90.3	118.6	148.2	**446.3**	**447.8**	**448.1**	**448.2**
XGBoost	2.2	4.0	6.4	9.4	47.4	82.7	110.3	136.0	448.3	449.7	450.0	450.2
LightGBM	1.4	3.2	6.0	9.6	46.9	81.6	114.1	139.4	577.8	579.1	579.5	579.7
CatBoost	0.8	1.9	3.4	5.2	**20.5**	**39.6**	**55.2**	**69.6**	557.8	558.5	558.6	558.7

Notes: Gaussian noise levels correspond to σ={0.05,0.10,0.15,0.20}×SD; impulse noise levels correspond to corruption rates p={10%,20%,30%,40%}; and calibration drift levels correspond to linear bias slopes d={0.5,1.0,1.5,2.0} mg/dL/h. Bold values indicate best performance for each metric.

**Table 7:** Temporal generalization gap: random vs. time-series CV $R^{2}$.

Model	Random $R^{2}$	Time-Series $R^{2}$	Gap $\Delta R^{2}$
Random Forest	0.922 ± 0.020	0.717 ± 0.073	0.205
AdaBoost	0.796 ± 0.050	0.678 ± 0.112	**0.118**
GBM	0.886 ± 0.025	**0.737 ± 0.082**	0.148
XGBoost	0.944 ± 0.014	0.686 ± 0.087	0.258
LightGBM	0.938 ± 0.013	0.710 ± 0.085	0.228
CatBoost	**0.957 ± 0.009**	0.720 ± 0.087	0.238

Note: Bold values indicate best performance for each metric.

**Table 8:** Feature importance stability on glucose data.

Model	Mean ρ	Std ρ
Random Forest	0.645 ± 0.034	0.097
AdaBoost	0.501 ± 0.040	0.094
GBM	0.533 ± 0.035	0.086
XGBoost	0.488 ± 0.024	0.085
LightGBM	0.579 ± 0.040	0.087
CatBoost	**0.679 ± 0.039**	**0.081**

Note: Bold values indicate best performance for each metric.

**Table 9:** PM_2.5_ forecasting results (24-h horizon; 12 Beijing stations).

Model	RMSE (μg/m^3^)	$R^{2}$	Train Time (s)
Random Forest	98.69 ± 12.71	0.048 ± 0.049	8.04 ± 0.99
AdaBoost	97.24 ± 11.62	0.072 ± 0.071	19.89 ± 1.17
GBM	99.78 ± 12.51	0.024 ± 0.082	33.73 ± 0.97
XGBoost	98.73 ± 12.28	0.044 ± 0.076	0.99 ± 0.08
LightGBM	98.77 ± 11.95	0.043 ± 0.071	1.72 ± 0.10
CatBoost	**97.02 ± 12.15**	**0.078 ± 0.061**	**0.88 ± 0.09**

Note: Bold values indicate best performance for each metric.

**Table 10:** Statistical significance tests for PM_2.5_ forecasting algorithms.

Friedman Omnibus Test						
Test Statistic	$\chi^{2}\left(5\right)=12.6$					
*p*-value	p=0.028					
Interpretation	Significant differences detected					
**Post hoc Nemenyi Pairwise Comparison ** ***p*** **-values**						
	Random Forest	AdaBoost	GBM	XGBoost	LightGBM	CatBoost
Random Forest	—	0.513	0.445	0.743	0.913	0.012 *
AdaBoost	0.513	—	0.156	0.743	0.445	0.064
GBM	0.445	0.156	—	0.275	0.513	0.001 **
XGBoost	0.743	0.743	0.275	—	0.663	0.029 *
LightGBM	0.913	0.445	0.513	0.663	—	0.009 **
CatBoost	0.012 *	0.064	0.001 **	0.029 *	0.009 **	—

Notes: Significance levels: ** *p* < 0.01 and * *p* < 0.05.

**Table 11:** RMSE degradation (%) under synthetic sensor perturbations for PM_2.5_ forecasting (24-h horizon).

Model	Gaussian Noise				Impulse Noise				Calibration Drift			
	**Lv.1**	**Lv.2**	**Lv.3**	**Lv.4**	**Lv.1**	**Lv.2**	**Lv.3**	**Lv.4**	**Lv.1**	**Lv.2**	**Lv.3**	**Lv.4**
Random Forest	0.0	0.2	0.6	1.2	1.8	3.6	5.3	7.7	102.7	104.1	103.9	103.7
AdaBoost	0.1	0.2	0.5	0.6	**0.7**	1.5	2.2	3.1	95.3	96.1	96.0	96.0
GBM	0.1	0.1	0.4	0.4	3.3	6.1	10.5	15.2	166.3	166.7	166.4	166.3
XGBoost	0.6	0.7	1.1	1.2	2.0	4.0	6.3	8.6	60.1	58.4	57.6	57.5
LightGBM	**−0.2**	**−0.3**	**−0.2**	0.1	1.2	3.1	4.3	5.7	28.8	26.2	25.4	25.2
CatBoost	−0.1	0.0	0.1	**0.0**	**0.7**	**1.2**	**1.8**	**1.6**	**12.1**	**12.0**	**11.9**	**12.0**

Notes: Gaussian perturbations follow σ={0.05,0.10,0.15,0.20}×SD(feature); impulse corruption replaces p={10%,20%,30%,40%} of feature values with random extremes; drift applies a cumulative bias with slope d={0.5,1.0,1.5,2.0}μg/m^3^/h. Bold values indicate best performance for each metric.

**Table 12:** PM_2.5_ temporal generalization gap.

Model	Random CV $R^{2}$	Time-Series $R^{2}$	Gap $\Delta R^{2}$
Random Forest	0.872 ± 0.008	−0.176 ± 0.068	1.048
AdaBoost	0.330 ± 0.042	**−0.086 ± 0.052**	**0.417**
GBM	0.714 ± 0.014	−0.268 ± 0.097	0.982
XGBoost	0.866 ± 0.011	−0.348 ± 0.098	1.214
LightGBM	0.866 ± 0.011	−0.240 ± 0.085	1.106
CatBoost	**0.885 ± 0.010**	−0.171 ± 0.080	1.056

Note: Bold values indicate best performance for each metric.

**Table 13:** PM_2.5_ feature-importance stability.

Model	Mean ρ	Std ρ
Random Forest	**0.779 ± 0.017**	0.090
AdaBoost	0.775 ± 0.029	**0.049**
GBM	0.726 ± 0.033	0.081
XGBoost	0.767 ± 0.015	0.069
LightGBM	0.771 ± 0.017	0.064
CatBoost	0.746 ± 0.025	0.084

Note: Bold values indicate best performance for each metric.

**Table 14:** Building-energy prediction results (1-h horizon averaged across 10 buildings).

Model	RMSE	MAE	$R^{2}$	MAPE (%)	Time (s)
Random Forest	6.62	4.23	0.591	9.6	57.73
AdaBoost	9.00	6.65	0.661	13.3	216.87
GBM	6.33	4.04	0.709	8.9	416.49
XGBoost	6.12	3.95	0.692	8.8	2.28
LightGBM	6.06	3.95	0.723	8.6	3.35
CatBoost	**5.67**	**3.82**	**0.764**	**8.3**	**3.41**

Note: Bold values indicate best performance for each metric.

**Table 15:** Statistical significance tests for building-energy prediction algorithms.

Friedman Omnibus Test						
Test Statistic	$\chi^{2}\left(5\right)=19.3$					
*p*-value	p=0.002					
Interpretation	Significant differences detected					
**Post hoc Nemenyi Pairwise Comparison ** ***p*** **-values**						
	Random Forest	AdaBoost	GBM	XGBoost	LightGBM	CatBoost
Random Forest	—	0.083	0.169	0.282	0.765	0.027 *
AdaBoost	0.083	—	0.002 **	0.005 **	0.042 *	<0.001 ***
GBM	0.169	0.002 **	—	0.765	0.282	0.403
XGBoost	0.282	0.005 **	0.765	—	0.437	0.256
LightGBM	0.765	0.042 *	0.282	0.437	—	0.056
CatBoost	0.027 *	<0.001 ***	**0.403**	**0.256**	**0.056**	—

Notes: Significance levels: *** *p* < 0.001, ** *p* < 0.01, and * *p* < 0.05. Bold values indicate best performance for each metric.

**Table 16:** RMSE degradation (%) under synthetic sensor perturbations for building-energy forecasting (1-h horizon). Lower values indicate higher robustness.

Model	Gaussian Noise				Impulse Noise				Calibration Drift			
	**Lv.1**	**Lv.2**	**Lv.3**	**Lv.4**	**Lv.1**	**Lv.2**	**Lv.3**	**Lv.4**	**Lv.1**	**Lv.2**	**Lv.3**	**Lv.4**
Random Forest	5.4	11.8	18.7	25.6	57.2	96.8	129.2	157.4	513.6	513.7	513.7	513.7
AdaBoost	**0.3**	**1.6**	**3.5**	**5.7**	**11.2**	**22.1**	**33.5**	**43.9**	**327.1**	**327.1**	**327.1**	**327.1**
GBM	4.1	9.4	15.7	22.6	51.6	89.6	121.1	148.7	551.3	551.3	551.4	551.4
XGBoost	10.7	14.7	20.3	27.0	53.2	90.7	122.9	151.3	536.9	537.0	537.1	537.1
LightGBM	4.8	9.5	15.6	22.8	53.6	91.5	122.9	151.0	585.7	585.8	585.8	585.8
CatBoost	2.6	5.8	10.2	14.5	29.3	53.1	73.9	93.2	488.3	488.4	488.4	488.4

Notes: Gaussian perturbations follow σ={0.05,0.10,0.15,0.20}×SD(feature); impulse corruption replaces p={10%,20%,30%,40%} of feature values with random extremes; drift applies a cumulative bias with slope d={0.5,1.0,1.5,2.0} kWh/h. Bold values indicate best performance for each metric.

**Table 17:** Energy temporal generalization gap: Random vs. time-series $R^{2}$. Gaps ($\Delta R^{2}$) reflect look-ahead bias from correlated temporal samples. Lower gaps indicate better temporal robustness.

Model	Random $R^{2}$	Time-Series $R^{2}$	Gap $\Delta R^{2}$
Random Forest	**0.986 ± 0.008**	0.287 ± 0.762	0.699
AdaBoost	0.903 ± 0.076	0.236 ± 0.794	0.667
GBM	0.967 ± 0.022	**0.340 ± 0.734**	**0.628**
XGBoost	0.985 ± 0.009	0.247 ± 0.832	0.738
LightGBM	0.984 ± 0.010	0.303 ± 0.689	0.681
CatBoost	**0.986 ± 0.009**	0.287 ± 0.749	0.699

Note: Bold values indicate best performance for each metric.

**Table 18:** Feature-importance stability across folds (Spearman ρ). High stability reflects consistent identification of key load drivers despite moderate overall $R^{2}$.

Model	Mean ρ	Std ρ
Random Forest	**0.822 ± 0.034**	0.063
AdaBoost	0.650 ± 0.065	0.095
GBM	0.708 ± 0.042	0.091
XGBoost	0.775 ± 0.039	0.072
LightGBM	0.756 ± 0.043	0.088
CatBoost	0.812 ± 0.058	**0.060**

Note: Bold values indicate best performance for each metric.

**Table 19:** Cross-domain statistical significance summary of algorithm comparisons.

Domain	Friedman Test	Best Algorithm	Significant Pairwise Differences (vs. Best)
Glucose (30-min)	$\chi^{2}\left(5\right)=22.2$, p<0.001	CatBoost (RMSE: 21.79 mg/dL)	AdaBoost (p<0.001), Random Forest (p=0.017), GBM (p=0.017), XGBoost (p=0.009). No sig. difference with LightGBM (p=0.064).
PM_2.5_ (24-h)	$\chi^{2}\left(5\right)=12.6$, p=0.028	CatBoost (RMSE: 97.02 μg/m^3^)	Random Forest (p=0.012), GBM (p=0.001), XGBoost (p=0.029), LightGBM (p=0.009). No sig. difference with AdaBoost (p=0.064).
Energy (1-h)	$\chi^{2}\left(5\right)=19.3$, p=0.002	CatBoost (RMSE: 5.67 kWh)	AdaBoost (p<0.001), Random Forest (p=0.027). No sig. differences with modern GBDT methods (XGBoost p=0.256, LightGBM p=0.056, GBM p=0.403).

Notes: All tests based on Friedman omnibus test followed by Nemenyi post hoc pairwise comparisons with significance threshold α=0.05. Sample sizes: glucose *n* = 6 patients, PM_2.5_ *n* = 12 stations, and energy *n* = 10 buildings.

**Table 20:** Unified cross-domain performance summary.

Domain	Model	RMSE	$R^{2}$	Time (s)	Temporal Gap	Stability (ρ)	Drift Robust. ^†^
Glucose	Random Forest	23.09	0.818	7.47	0.205	0.645	573%
	AdaBoost	26.55	0.762	18.62	**0.118**	0.501	479%
	GBM	22.92	0.821	29.93	0.148	0.533	**448%**
	XGBoost	23.09	0.819	1.97	0.258	0.488	450%
	LightGBM	22.82	0.823	2.04	0.228	0.579	580%
	CatBoost	**21.79**	**0.838**	**1.69**	0.238	**0.679**	559%
PM_2.5_	Random Forest	98.69	0.048	8.04	1.048	**0.779**	104%
	AdaBoost	97.24	0.072	19.89	**0.417**	0.775	96%
	GBM	99.78	0.024	33.73	0.982	0.726	166%
	XGBoost	98.73	0.044	0.99	1.214	0.767	58%
	LightGBM	98.77	0.043	1.72	1.106	0.771	25%
	CatBoost	**97.02**	**0.078**	**0.88**	1.056	0.746	**12%**
Energy	Random Forest	6.62	0.591	57.73	0.699	**0.822**	514%
	AdaBoost	9.00	0.661	216.87	0.667	0.650	**327%**
	GBM	6.33	0.709	416.49	**0.628**	0.708	551%
	XGBoost	6.12	0.692	**2.28**	0.738	0.775	537%
	LightGBM	6.06	0.723	3.35	0.681	0.756	586%
	CatBoost	**5.67**	**0.764**	3.41	0.699	0.812	488%

Notes: ^†^ Drift robustness: RMSE degradation (%) at maximum drift intensity. Lower is better. RMSE units: glucose (mg/dL), PM_2.5_ (μg/m^3^), and energy (kWh). Bold values indicate best performance for each metric.

**Table 21:** Cross-domain robustness summary: RMSE degradation (%) at maximum perturbation intensity. Lower values indicate higher robustness.

Domain	Model	Gaussian (Max)	Impulse (Max)	Drift (Max)
Glucose	Random Forest	9.1	149.2	573.3
	AdaBoost	**4.9**	89.7	479.0
	GBM	10.3	148.2	**448.2**
	XGBoost	9.4	136.0	450.2
	LightGBM	9.6	139.4	579.7
	CatBoost	5.2	**69.6**	558.7
PM_2.5_	Random Forest	1.2	7.7	103.7
	AdaBoost	0.6	3.1	96.0
	GBM	0.4	15.2	166.3
	XGBoost	1.2	8.6	57.5
	LightGBM	0.1	5.7	25.2
	CatBoost	**0.0**	**1.6**	**12.0**
Energy	Random Forest	25.6	157.4	513.7
	AdaBoost	**5.7**	**43.9**	**327.1**
	GBM	22.6	148.7	551.4
	XGBoost	27.0	151.3	537.1
	LightGBM	22.8	151.0	585.8
	CatBoost	14.5	93.2	488.4

Note: Bold values indicate best performance for each metric.

## Data Availability

The datasets analyzed in this study are publicly available from the following sources: (1) Glucose monitoring data from the OhioT1DM Dataset (https://webpages.charlotte.edu/rbunescu/data/ohiot1dm/OhioT1DM-dataset.html), (2) PM_2.5_ forecasting data from the Beijing Multi-Site Air Quality Dataset (https://archive.ics.uci.edu/dataset/501/beijing+multi+site+air+quality+data), and (3) Building energy consumption data from the Building Data Genome Project 2.0 (https://service.tib.eu/ldmservice/en/dataset/building-data-genome-2-0–bdg2-) (All accessed on 25 November 2025).
